# Metal-Mediated Addition of N-Nucleophiles to Isocyanides: Mechanistic Aspects

**DOI:** 10.3390/molecules22071141

**Published:** 2017-07-08

**Authors:** Maxim L. Kuznetsov, Vadim Yu. Kukushkin

**Affiliations:** 1 Centro de Química Estrutural, Instituto Superior Técnico, Universidade de Lisboa, Av. Rovisco Pais, Lisbon 1049-001, Portugal; 2 International Group on Organometallic Chemistry, Institute of Chemistry, Saint Petersburg State University, Universitetskaya Nab., 7/9, Saint Petersburg 199034, Russia; v.kukushkin@spbu.ru

**Keywords:** isocyanides, nitriles, nucleophilic addition, DFT calculations, activation of small molecules, reaction mechanism, carbenes

## Abstract

Despite the long history of the investigation of nucleophilic addition to metal-bound isocyanides, some important aspects of the reaction mechanism remain unclear even for the simplest systems. In this work, the addition of the sp^3^-N, sp^2^-N, and mixed sp^2^/sp^3^-N nucleophiles (i.e., HNMe_2_, HN=CPh_2_, and H_2_N–N=CPh_2_, respectively) to isocyanides C≡NR coordinated to the platinum(II) centers in the complexes *cis*-[Pt(C≡NCy)(2-pyz)(dppe)]^+^ (2-pyz = 2-pyrazyl, dmpe = Me_2_PCH_2_CH_2_PMe_2_) and *cis*-[PtCl_2_(C≡NXyl)(C≡NMe)] was studied in detail by theoretical (DFT) methods. The mechanism of these reactions is stepwise associative rather than concerted and it includes the addition of a nucleophile to the isocyanide C atom, deprotonation of the nucleophilic moiety in the resulting intermediate, and protonation of the isocyanide N atom to give the final product. The calculated activation energy (ΔG^≠^) of all reactions is in the range of 19.8–22.4 kcal/mol.

## 1. Introduction

**Scheme 1:**
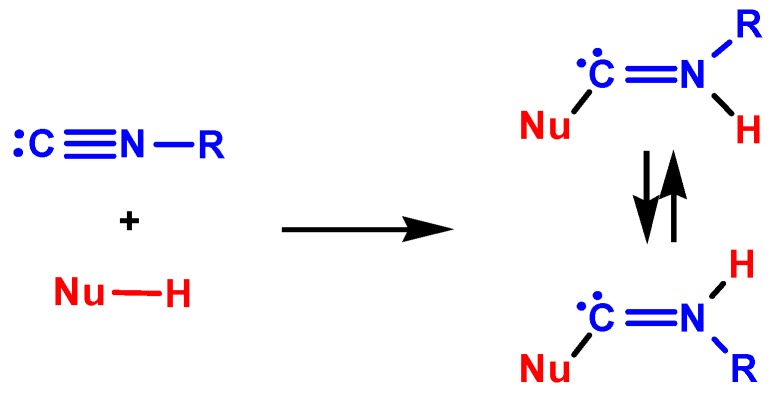
Addition of nucleophile Nu–H to isocyanides (formal charges are omitted).

Isocyanides (C≡NR) are highly demanding reagents toward materials with valuable properties [1,2,3] and they have a broad application in multicomponent [4,5,6,7,8,9,10,11,12] and insertion [4,13,14,15,16,17,18] reactions, catalysis [4,13,14,19,20,21,22], and polymer chemistry [15]. One of the most important types of the reactivity involving isocyanides is nucleophilic addition (NA) at the carbon atom of the C≡N group (Scheme 1). This reaction is one of the most efficient and promising methods for the synthesis of heteroatom stabilized carbenes—species of superior importance exhibiting valuable biological [23,24], catalytic [19,25,26,27,28,29,30,31,32,33,34,35,36,37,38,39], and photophysical [40,41,42,43] properties. These reactions typically require the presence of a metal center to activate C≡NR. The first metal-mediated reaction of C≡NR species was observed more than 100 years ago by Tschugajeff (Chugaev) and Skanawy-Grigorjewa [44,45]. As a result of the interaction between K_2_[PtCl_4_] and C≡NMe and the following addition of hydrazine, the first complex of a heteroatom-stabilized carbene was obtained [46,47,48,49]. Since that time, the metal-mediated addition of the monofunctional O- [50,51,52,53,54], N- [34,35,36,37,41,43,51,52,53,54,55,56,57,58,59,60,61,62,63,64,65,66,67,68,69,70], and other [71,72,73,74,75,76,77,78] nucleophiles as well as bifunctional nucleophiles [27,34,53,62,64,79,80,81,82,83,84,85,86,87,88,89] to isocyanides was broadly investigated [1,2,3,90,91].

The N–H nucleophiles with the sp^3^ type of the nitrogen orbital hybridization (e.g., amines and hydrazines) are among the most common reagents used in this reaction, and their addition to C≡NR has been intensively exploring over several decades [34,35,36,37,41,43,51,52,53,54,55,56,57,58,59,60,61,62,63,64,65,66,67,68,69,70,92], including the experimental kinetic studies [93,94]. However, surprisingly, despite the long history of these researches, some important aspects of the reaction mechanism still remain unknown, even for the simplest systems. First, the experiments indicate the negative activation entropy and, therefore, the associative general type of the mechanism for the NA of amines to metal-bound isocyanides and the bimolecular rate limiting step of this reaction. Meanwhile, experimental results cannot discriminate the concerted (I_a_) and stepwise (A) mechanism of the nucleophilic attack—both of them exhibiting the associative activation mode [93,94]. Second, the addition of N–H nucleophiles of the sp^2^-N type (imines [95]) or the mixed sp^2^/sp^3^-N type, such as hydrazones [32], to metal-bound isocyanides was reported only recently and, to the best of our knowledge, no mechanistic studies of these processes were undertaken. Third, hydrazones bear two nucleophilic centers which may competitively participate in the NA to C≡NR. However, in contrast to the sp^3^-N bifunctional nucleophiles, no analysis of the regioselectivity from the mechanistic point of view was done in this case.

The main goal of this work is to fill this gap in the understanding of the intimate mechanism of the NA of N–H nucleophiles to metal-bound isocyanides. The reactions of three N–H nucleophiles of the sp^3^-N, sp^2^-N, and sp^2^/sp^3^-N type (HNR_2_, HN=CPh_2_, and H_2_N–N=CPh_2_, respectively) with the complexes *cis*-[Pt(C≡NCy)(2-pyz)(dppe)]^+^ (**I**, 2-pyz = 2-pyrazyl, dppe = Ph_2_PCH_2_CH_2_PPh_2_) and *cis*-[PtCl_2_(C≡NXyl)_2_] (**II**) for which experimental data are available [32,93,94,95] were selected for this study. In the computational models, the dppe ligand was substituted by the dmpe ligand (dmpe = Me_2_PCH_2_CH_2_PMe_2_) and one of the C≡NXyl ligands in **II** which remains unreacted was replaced for the C≡NMe ligand.

## 2. Computational Details

The full geometry optimization of all structures and transition states (TSs) was carried out at the DFT level of theory by using the M06L functional [96] with the help of the Gaussian 09 [97] program package. No symmetry operations were applied. The geometry optimization was carried out by using a quasi-relativistic Stuttgart pseudopotential that describes 60 core electrons and the appropriate contracted basis set (8s7p6d)/[6s5p3d] [98] for the platinum atom and the 6-31G* basis set for other atoms. Single-point calculations were then performed on the basis of the equilibrium geometries found by using the 6-311+G** basis set for non-metal atoms. The solvent effects were taken into account in both optimization and single-point calculations using the SMD model [99] with dichloroethane (for reaction of complex **1**) or chloroform (for reactions of complex **2**) as solvents. If not stated otherwise, the energies discussed below are Gibbs free energies G(6-311+G**) calculated as G(6-311+G**) = E(6-311+G**) – E(6-31G*) + G(6-31G*) where the basis set used is indicated.

The Hessian matrix was calculated analytically for the optimized structures to prove the location of correct minima (no imaginary frequencies) or saddle points (only one imaginary frequency), and to estimate the thermodynamic parameters, with the latter calculated at 25 °C. The nature of all transition states was investigated by analysis of the vectors associated with the imaginary frequency and by the calculations of the intrinsic reaction coordinates (IRC) by using the method developed by Gonzalez and Schlegel [100,101,102]. The Wiberg bond indices [103] and atomic charges were computed by using the natural-bond orbital (NBO) partitioning scheme [104].

## 3. Results and Discussion

### 3.1. Mechanisms of NA to Isocyanides

**Scheme 2:**
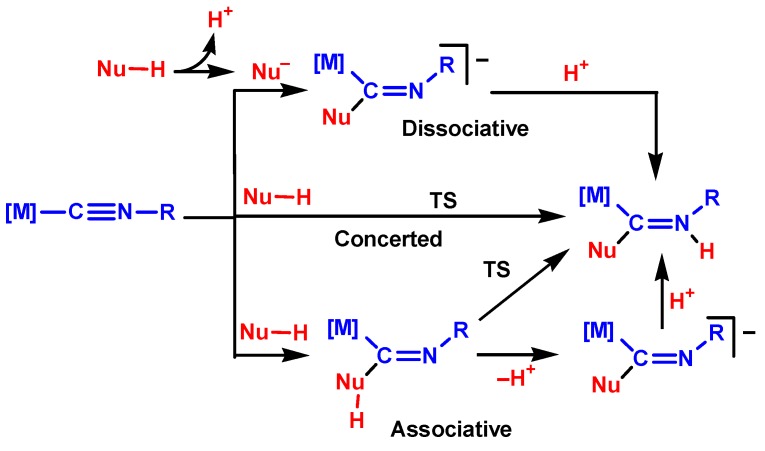
Mechanisms of nucleophilic addition to metal-bound isocyanides (only one isomeric pathway is shown, formal charges are omitted).

**Figure 1:**
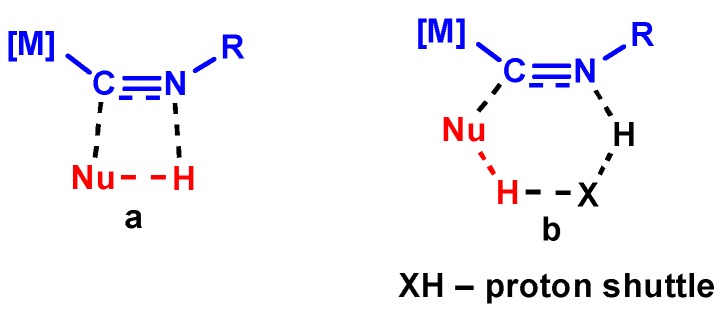
Types of transition state of the concerted nucleophilic addition to metal-bound isocyanides.

There are three main types of the mechanisms for NA to isocyanides, i.e., concerted, dissociative, and associative (Scheme 2). The concerted mechanism involves one-step attack of a nucleophile (NuH) at the C atom of the C≡N group accompanied by simultaneous H-transfer to the isocyanide N atom. This mechanism includes one transition state which may be either 4-membered (**a**) or 6-membered (**b**) (Figure 1). In the latter case, either second molecule of the reagent or a solvent molecule directly participates in the formation of TS and plays the role of an H-transfer promoter.

The dissociative mechanism includes the initial deprotonation of NuH, addition of the deprotonated fragment to the isocyanide C atom, and protonation of the isocyanide N atom. The deprotonation of NuH may be assisted by any base from the reaction mixture, e.g., water molecules often coming as moisture with organic solvents, nucleophilic additives or ligands or nucleophile itself.

The associative mechanism starts with the nucleophilic attack of NuH at the isocyanide C atom to give an acyclic intermediate which may be converted into the final product as a result of the proton transfer which, in turn, may be either concerted or stepwise.

### 3.2. Reaction of the sp^3^-N Nucleophiles. Comparison with the Experimental Kinetic Data

The simplest representatives of the N–H nucleophiles featuring the sp^3^ hybridization of the nitrogen atom orbitals are amines. In this section, the reaction between dimethylamine HNMe_2_ and the isocyanide Pt complex *cis*-[Pt(C≡NCy)(2-pyz)(dmpe)]^+^ (**1**) is discussed. As was indicated in the Introduction, experimental kinetic data are available for the similar system HNEt_2_ + *cis*-[Pt(C≡NCy)(2-pyz)(dppe)]^+^. Therefore, comparison of the calculated and experimental kinetic parameters allows the testing of the computational method and model used in this work. Additionally, some mechanistic features of this simple process still remain uncertain (see Introduction).

#### 3.2.1. Concerted Mechanism

The calculations revealed that the concerted mechanism is not realized for this process. Indeed, an extensive search of the potential energy surface (PES) indicated that there is no minimum corresponding to any of the TSs of the concerted mechanism. All attempts of its location led to TSs of the stepwise pathways discussed below. This result is different from that found previously for NA to nitriles N≡CR for which both 4-membered and 6-membered cyclic TSs of the concerted NA were located [105].

#### 3.2.2. Dissociative Mechanism

**Figure 2:**
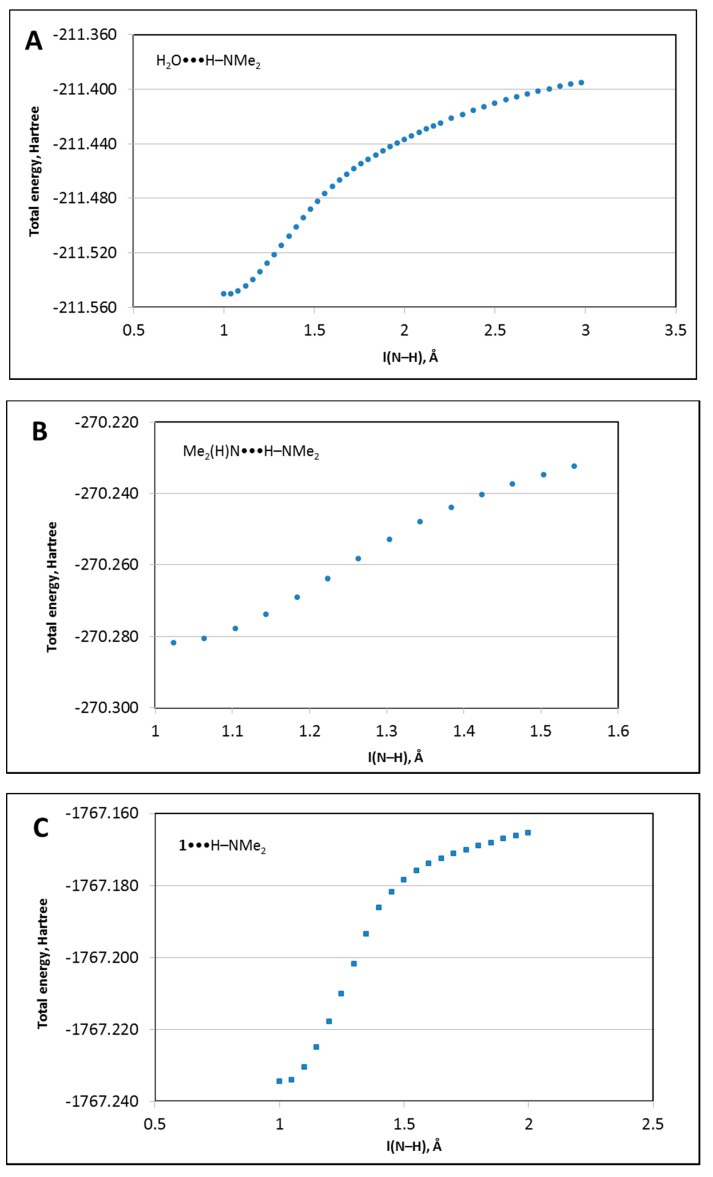
Total energy vs. the N–H distance in the systems H_2_O···H–NMe_2_ (**A**), Me_2_(H)N···H–NMe_2_ (**B**), and **1**···H–NMe_2_ (**C**).

Within this mechanism, H_2_O, HNMe_2_, and the pyrazine ligand in **1** were considered as bases for the initial proton abstraction from HNMe_2_ (reactions 1–3). The PCM-based solvation models often fail to estimate correctly solvent effects for processes, in which the number of species with the same charge is not preserved in the course of the reaction. Therefore, straightforward calculations of the ΔG_s_ values of reactions 1–3 as a difference ΣG_s_(products) – ΣG_s_(reactants) is not appropriate. To study the possibility of realization of the dissociative mechanism, a scan of the potential energy surface with the fixed N–H bond of HNMe_2_ and relaxed other bonds was undertaken for the systems H_2_O···H–NMe_2_, Me_2_(H)N···H–NMe_2_, and **1**···H–NMe_2_. The energy of the systems monotonously increases upon enhancement of the NH internuclear distance, demonstrating the absence of any TS for these processes (Figure 2). The lowest limits of the activation energy E_a,s_ estimated from the energy curves are 97.6, 30.9, and 43.3 kcal/mol for reactions 1–3, respectively. Taking into account that the entropic factor is insignificant for these processes, the estimates for the ΔG_s_^≠^ limits should also be close to these values. Thus, the dissociative mechanism may be ruled out due to inefficient deprotonation of the nucleophile.

HNMe_2_ + H_2_O → NMe_2_^−^ + H_3_O^+^ (1)

HNMe_2_ + HNMe_2_ → NMe_2_^−^ + H_2_NMe_2_^+^ (2)

HNMe_2_ + *cis*-[Pt(CNCy)(2-pyz)(dmpe)]^+^ → NMe_2_^−^ + *cis*-[Pt(CNCy)(2-pyzH)(dmpe)]^2+^ (3)

#### 3.2.3. Associative Mechanism

**Figure 3:**
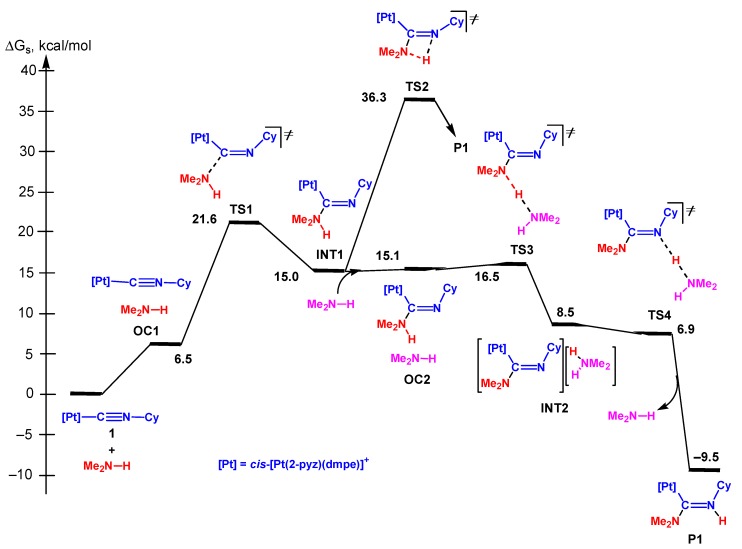
Energy profile for the associative mechanism of the nucleophilic addition of HNMe_2_ to *cis*-[Pt(C≡NCy)(2-pyz)(dmpe)]^+^.

This pathway starts with the formation of the orientation van der Waals complexes **OC1** between HNMe_2_ and **1** (Figure 3). The process is exothermic by –5.8 kcal/mol but endoergonic by 6.5 kcal/mol due to unfavorable entropic factor. **OC1** transforms into transition state **TS1** corresponding to the formation of the CN bond between reactant molecules and then into the acyclic intermediate **INT1**.

The proton transfer **INT1** → **P1** may occur either in a concerted or in a stepwise manner. Four-membered **TS2** of the concerted H-transfer was found, whereas no 6-membered transition state of this route was located. However, a high energy of **TS2** relative to the level of initial reactants (36.3 kcal/mol) indicates that the pathway based on this TS is not realized.

The stepwise H-transfer in **INT1** includes (*i*) energetically neutral formation of the orientation complex **OC2** between **INT1** and the second HNMe_2_ molecule, (*ii*) deprotonation of **INT1** with HNMe_2_ via **TS3** to give intermediate **INT2** with very low activation barrier of 1.4 kcal/mol relative to **OC2**, and (*iii*) protonation of the isocyanide N atom by H_2_NMe_2_^+^ via **TS4** affording the final product **P1**. The Gibbs free energy of **TS4** is lower than that of **INT2**. This phenomenon is accounted for by the fact that total energy of the system was minimized upon the geometry optimization rather than Gibbs free energy. Indeed, in terms of total energy, the E_s_ value of **TS2** is higher than that of **INT2** by 0.7 kcal/mol.

Thus, the computational results indicate that the most plausible mechanism of the reaction between HNMe_2_ and **1** is of the stepwise associative type. The first nucleophilic attack is the rate limiting step, while the proton transfer in **INT1** occurs very fast with assistance of the second nucleophile molecule. These theoretical predictions are in agreement with strongly negative experimental activation entropy value of –43 ± 3 e.u. for the reaction HNEt_2_ + **I**. The calculated enthalpy and Gibbs free energy of activation (6.9 and 21.6 kcal/mol) are in good agreement with the corresponding experimental values 7.0 ± 0.8 and 19.8 ± 1.7 kcal/mol [93], thus validating the used computational method and model.

### 3.3. Reactions of the sp^2^-N and sp^2^/sp^3^-N Nucleophiles

In this section, reactions of the NH nucleophiles with predominantly sp^2^ and intermediate between sp^2^ and sp^3^ types of the nitrogen orbital hybridization are discussed. Imines HN=CR_2_ are the simplest representatives of the first group of NuH, whereas hydrazones R’HN–N=CR_2_ are typical examples of the second group. The natural bond orbital (NBO) analysis of benzophenone hydrazone H_2_N–N=CPh_2_ shows that hybridization of the amino nitrogen orbital forming the σ-NBO with the central N atom is sp^2.5^. Additions of benzophenone imine HN=CPh_2_ and benzophenone hydrazone H_2_N–N=CPh_2_ to the xylylisocyanide ligand in the complex *cis*-[PtCl_2_(CNMe)(CNXyl)] (**2**) were calculated as examples of these types of NA.

#### 3.3.1. Concerted and Dissociative Mechanisms

Similarly to the previous case, no transition states of the concerted NA were found for the reactions of **2** with both the imine and the hydrazone. On the other hand, the lowest limits of the activation energy E_a,s_ found for the proton dissociation from HN=CPh_2_ or H_2_NN=CPh_2_ are in the range of 29.0–65.3 kcal/mol (reactions 4–7) (Figures S1 and S2 in Supplementary Material). The hydrazone appears to be a stronger acid than imine and a stronger base than water due to ambivalent character of this reagent. Thus, the dissociative pathway could be realized, in principle, only for the reaction of H_2_N–N=CPh_2_ with **2** when another molecule of hydrazone plays the role of a base, while for the imine HN=CPh_2_ this mechanism may be excluded.

HN=CPh_2_ + H_2_O → N=CPh_2_^−^ + H_3_O^+^ E_a,s_ ≥ 65.3 kcal/mol (4)

HN=CPh_2_ + HN=CPh_2_ → N=CPh_2_^−^ + H_2_N=CPh_2_^+^ E_a,s_ ≥ 35.6 kcal/mol (5)

H_2_N–N=CPh_2_ + H_2_O → HN–N=CPh_2_^−^ + H_3_O^+^ E_a,s_ ≥ 57.8 kcal/mol (6)

H_2_N–N=CPh_2_ + H_2_N–N=CPh_2_ → HN–N=CPh_2_^−^ + H_2_N–N(H)=CPh_2_^+^ E_a,s_ ≥ 29.0 kcal/mol (7)

#### 3.3.2 Associative Mechanism

**Figure 4:**
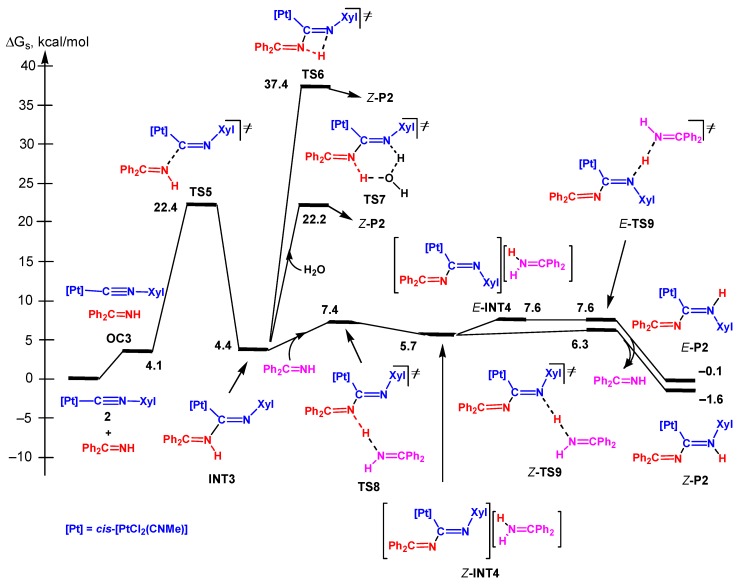
Energy profile for the associative mechanism of nucleophilic addition of HN=CPh_2_ to the C≡NXyl ligand in *cis*-[PtCl_2_(C≡NMe)(C≡NXyl)].

**Figure 5:**
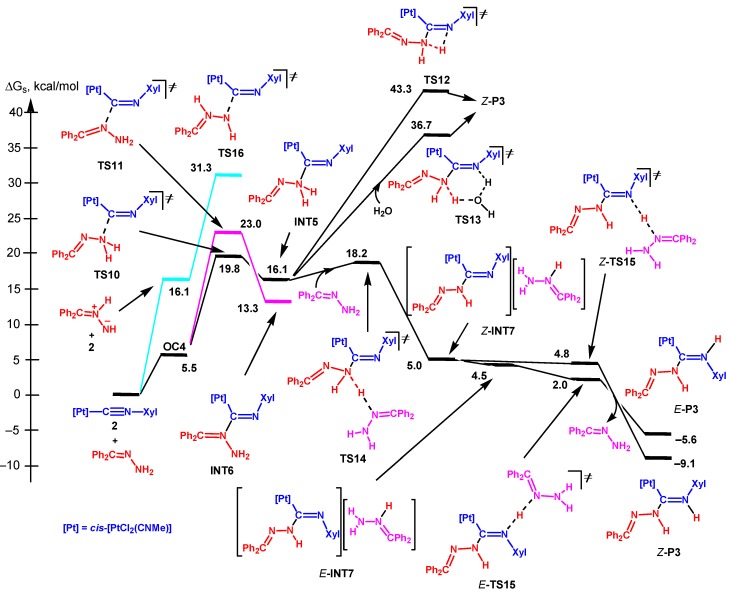
Energy profile for the associative mechanism of nucleophilic addition of H_2_N–N=CPh_2_ to the C≡NXyl ligand in *cis*-[PtCl_2_(C≡NMe)(C≡NXyl)].

This pathway includes the formation of orientation complexes **OC3** and **OC4**, transition states associated with the C–N bond creation (**TS5** and **TS10**), and intermediates **INT3** and **INT5** (Figure 4 and Figure 5). The further H-transfers **INT3** → **P2** and **INT5** → **P3** in these intermediates occur in a stepwise manner. The second molecule of NuH abstracts a proton from **INT3** and **INT5** to give deprotonated species **INT4** and **INT7**. In these complexes, a nitrogen inversion in the bent isocyanide ligand should occur easily. Hence, there are two possible channels for the protonation of **INT4** and **INT7** leading either to *Z*-isomer or to *E*-isomer of the final products **P2** and **P3**. The overall activation barriers for this pathway are 22.4 and 19.8 kcal/mol for the reactions of the imine and the hydrazone, respectively.

Besides, for these nucleophiles, both 4-and 6-membered cyclic transition states **TS6**, **TS7**, **TS12**, and **TS13** of the concerted H-transfer were also located. However, this mechanism is significantly less favorable than the stepwise proton shift (Figure 4 and Figure 5).

#### 3.3.3. Nucleophilic Addition by the Imino N Atom of Hydrazone

The hydrazone molecule H_2_N–N=CPh_2_ has two nucleophilic centers, i.e., the amino and imino nitrogen atoms. Hence, the hydrazone may also attack the isocyanide carbon atom by another, imino nitrogen atom. The amino N atom has significantly higher negative effective NBO atomic charge than the imino N atom (–0.67 vs. –0.27 e). However, the imino N atom is a stronger nucleophile from the thermodynamic point of view. Among two protonated structures, H_3_N–N=CPh_2_^+^ and H_2_N–N(H)=CPh_2_^+^, the latter is 5.9 kcal/mol more stable than the former. The corresponding intermediate **INT6** was also found to be more stable than **INT5** by 2.8 kcal/mol (Figure 5). However, transition state **TS11** leading to **INT6** is higher in energy than **TS10** by 3.2 kcal/mol that correlates with the atomic charge distribution—one of the principal factors determining the kinetic of nucleophilic additions. Thus, the participation of the imino N atom in the reaction is less favorable and, therefore, other steps of this mechanism were not calculated.

#### 3.3.4. Mechanism Involving the Dipole Tautomeric form of Hydrazone

The H-transfer from the amino N atom to the imino nitrogen in the H_2_N–N=CPh_2_ molecule leads to the dipole tautomeric form HN^–^–N^+^(H)=CPh_2_ and thus should enhance the nucleophilic properties of the terminal nitrogen atom. Indeed, the activation barrier for the NA of HN^–^–N^+^(H)=CPh_2_ to **2** is 15.3 kcal/mol vs. 19.8 kcal/mol for the H_2_N–N=CPh_2_ form. However, the tautomerization is very endoergonic process (by 16.1 kcal/mol) and, as a result, the overall activation barrier for this mechanism is much higher (31.3 kcal/mol, Figure 5).

#### 3.3.5. Analysis of the Energy Profiles

Analysis of the energy profiles for these reactions indicates the following (Figure 3, Figure 4 and Figure 5). First, the most plausible mechanism is of the stepwise associative type. Second, in the case of hydrazone, the amino nitrogen atom participates in the reaction rather than the imino N atom despite the latter is a stronger nucleophile than the former from the thermodynamic point of view. Third, the rate limiting step of the whole process is the initial nucleophilic addition via **TS5** and **TS10**, while the further H-transfer step requires very low activation barrier.

Fourth, a distinctive feature of the reaction of the imine with **2** is much higher relative stability of **INT3** and **TS8** (sp^2^ N nucleophile) compared to **INT5** and **TS14** (sp^2^/sp^3^ N nucleophile). **INT3** and **TS8** are only slightly higher in energy than the initial reactants level (by 4.4 and 7.4 kcal/mol), whereas the energies of **INT5** and **TS14** are comparable with those of the rate limiting **TS10** (16.1 and 18.2 kcal/mol). Such a difference is accounted for by an extra stabilization of **INT3** and **TS8** due to the conjugation in the N=C–N=C fragments and delocalization of electron density. Indeed, the calculated Wiberg bond indices of two C=N bonds in **INT3** are lower than those in **INT5** (1.76 and 1.43 vs. 1.86 and 1.70), and the Wiberg index of the C–N bond is higher in **INT3** than in **INT5** (0.96 vs. 0.79). Correspondingly, the C–N bond is noticeably shorter in **INT3** than in **INT5** (1.45 Å vs. 1.56 Å).

Fifth, the *Z*-isomer of the final products *Z*-**P2** and *Z*-**P3** is more thermodynamically stable than the corresponding *E*-isomer (by 1.4–3.5 kcal/mol). The formation of *Z*-**P2** is both kinetically and thermodynamically controlled. In contrast, for the reaction with hydrazone, *Z*-**P3** is the thermodynamic product, while *E*-**P3** is the kinetic one. There is no experimental data about the structure of the **P2** type product. Meanwhile, the *E*-**P3** type product was experimentally isolated and its structure was determined by the X-ray diffraction. A low activation barrier of the *E*-**P3** to *Z*-**P3** isomerization (10.6 kcal/mol) should permit this process in solution. Therefore, the experimental structural data for *E*-**P3** may be accounted for by an effect of the crystal packing which stabilizes the *E*-configuration in the solid state due to favorable intermolecular interactions.

Sixth, ΔG_s_ of formation of the reaction product with the imine, *Z*-**P2**, is only slightly negative (−1.6 kcal/mol), whereas the final reaction product with the hydrazone, *Z*-**P3**, is clearly exoergonic (by −9.1 kcal/mol). This correlates with the experimental detection of unreacted isocyanide in the reaction mixture *cis*-[PtCl_2_(CNXyl)_2_] + HN=CPh_2_ under synthesis conditions and with the fact that **P2** was not isolated in solid state [95], while the product *E*-**P3** was successfully isolated and characterized [32].

**Figure 6:**
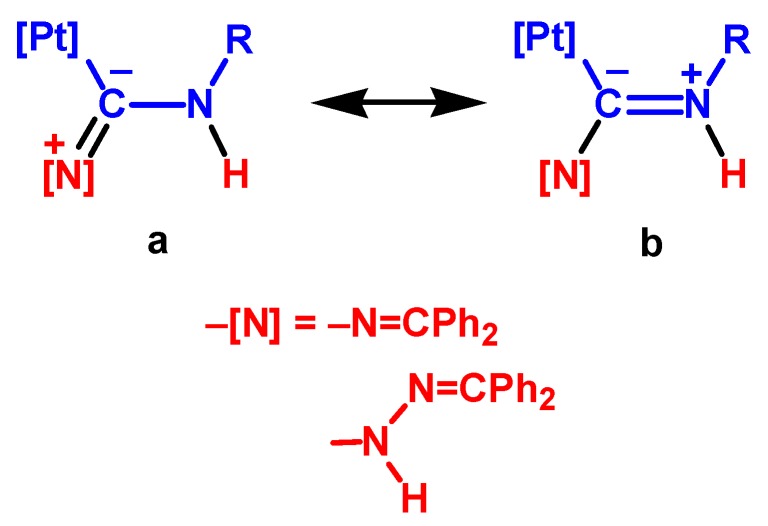
Resonance structures of the nucleophilic addition products.

Seventh, the distribution of the Wiberg bond indices in the reaction products **P2** and **P3** indicates that these complexes has an electronic structure intermediate between two resonance forms **a** and **b** (Figure 6).

Eighth, the overall activation energies of all three reactions discussed above are similar (19.8–22.4 kcal/mol), demonstrating the low effect of the nitrogen orbital hybridization on the reactivity of the NH-nucleophiles. The kinetic information obtained here for the reactions with imines and hydrazones is particularly important since no experimental kinetic data are available for these processes.

## 4. Final Remarks

Metal-mediated nucleophilic addition to isocyanides is a very important route toward highly demanding heteroatom-stabilized carbene species. Despite a long history of the investigation of these processes, some important information about the intimate reaction mechanism remained unclear. In this work, a theoretical DFT study has been undertaken with the aim to fill this gap for the reactions of three types of the NH-nucleophiles (i.e., sp^3^-N, sp^2^-N, and sp^2^/sp^3^-N types) with Pt-bound C≡NR. The calculations demonstrated that in all cases the mechanism is stepwise of the associative type (A), and it includes addition of a nucleophile to the isocyanide C atom, deprotonation of the resulting intermediate (e.g., by the second molecule of a nucleophile or by another base from the reaction mixture), and the protonation of the isocyanide N atom to give the final product. The nucleophilic addition step is rate limiting for the whole process. The overall activation energy weakly depends on the nucleophile nature and varies from 19.8 to 22.4 kcal/mol. To our knowledge, this is the first estimate of the kinetic parameters for the metal-mediated NA of the sp^2^-N and sp^2^/sp^3^-N nucleophiles to isocyanides. Alternative mechanisms (i.e. concerted and dissociative) were found to be not feasible. In the case of hydrazones, which are bifunctional nucleophiles, participation of the amine N atom is more preferable than the imine N atom, and the regioselectivity of this reaction is charge-driven.

## Supplementary Materials

The following are available online at www.mdpi.com/1420-3049/22/7/1141/s1, Figure S1: total energy vs. the N–H distance in the systems H2O···H–N=CPh2, Ph2C=(H)N···H–N=CPh2, Figure S2: total energy vs. the N–H distance in the systems H_2_O···H–N(H)–N=CPh_2_, Ph_2_C=(NH_2_)N···H–N(H)–N=CPh_2_, Table S1: calculated total energies, enthalpies, Gibbs free energies, and entropies, Table S2: Cartesian atomic coordinates of the equilibrium structures.
