# Supplementary material for: Metal-Mediated Addition of N-Nucleophiles to Isocyanides: Mechanistic Aspects
Source: Molecules. 2017 Jul 8;22(7):1141. doi: 10.3390/molecules22071141 (PMC6152363; doi:10.3390/molecules22071141)
Supplement: Supplementary file 1 [file molecules-22-01141-s001.pdf]

Metal-mediated addition of N-nucleophiles to isocyanides: mechanistic aspects

Maxim L. Kuznetsov [1,2,*] and Vadim Yu. Kukushkin[2]

[1] Centro de Química Estrutural, Instituto Superior Técnico, Universidade de Lisboa, Av. Rovisco Pais, 1049-001 Lisbon, Portugal; max@mail.ist.utl.pt
[2] International Group on Organometallic Chemistry, Institute of Chemistry, Saint Petersburg State University, 199034, Universitetskaya Nab., 7/9, Saint Petersburg, Russian Federation; kukushkin@VK2100.spb.edu

Supplementary Material

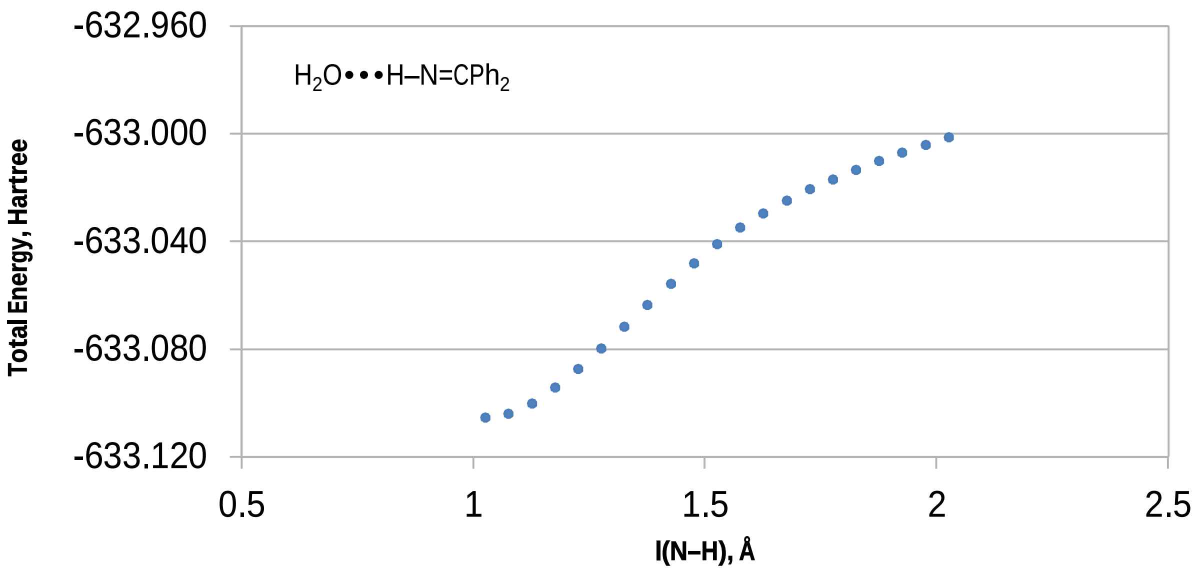

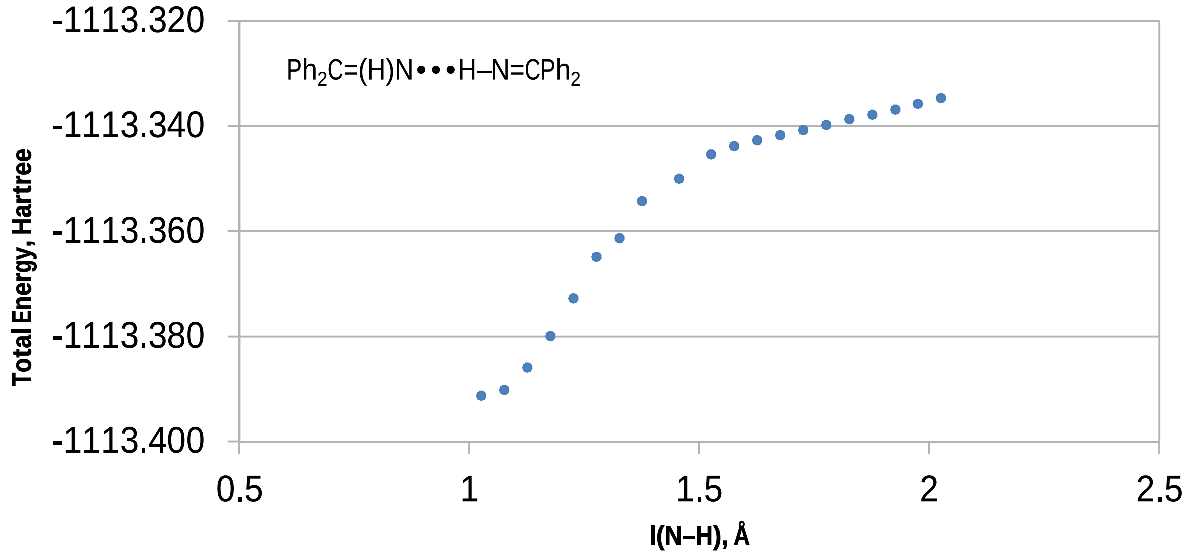

Figure S1. Total energy *vs.* the N–H distance in the systems H_2O•••H–N=CPh_2, Ph_2C=(H)N•••H–N=CPh_2.

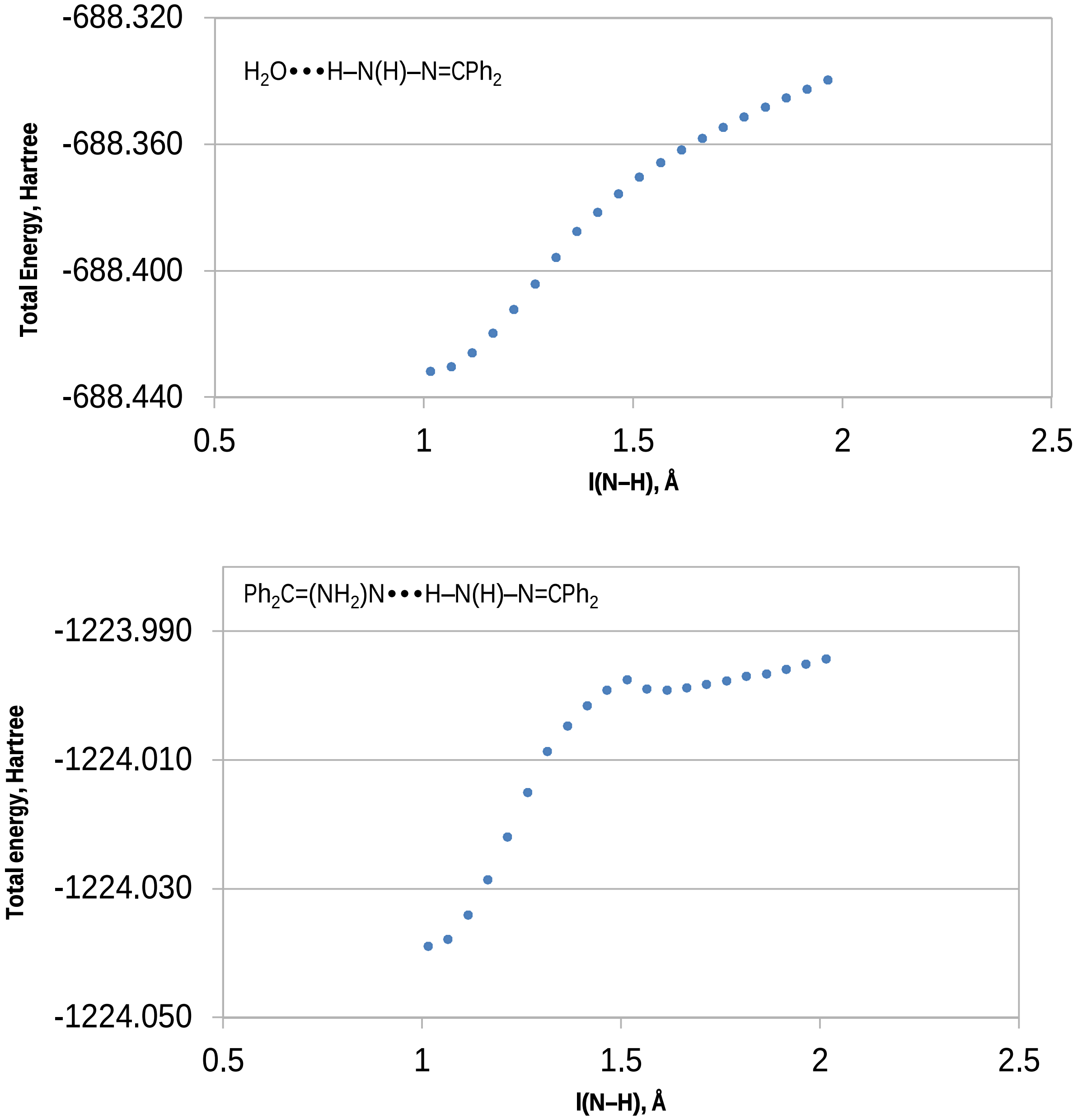

Figure S2. Total energy *vs.* the N–H distance in the systems H_2O•••H–N(H)–N=CPh_2, Ph_2C=(NH_2)N•••H–N(H)–N=CPh_2.

Table S1. Calculated total energies, enthalpies, Gibbs free energies (in Hartree) and entropies (in cal/mol•K).

	E (6-31G*)	E (6-311+G**)	H (6-31G*)	S	G (6-31G*)	G (6-311+G**)
H_2O	-76.406966	-76.448171	-76.381774	46.51	-76.403871	-76.445076
Me_2NH	-135.137153	-135.184312	-135.038881	64.66	-135.069602	-135.116761
**1**	-1632.089309	-1632.361948	-1631.608656	187.32	-1631.697658	-1631.970297
**OC1**	-1767.239164	-1767.557389	-1766.658379	210.76	-1766.758516	-1767.076741
**TS1**	-1767.219255	-1767.535853	-1766.639723	202.69	-1766.736028	-1767.052626
**INT1**	-1767.235546	-1767.551801	-1766.653061	197.51	-1766.746902	-1767.063157
**TS2**	-1767.190945	-1767.508891	-1766.615232	201.99	-1766.711205	-1767.029151
**OC2**	-1902.393746	-1902.754665	-1901.711611	225.69	-1901.818842	-1902.179761
**TS3**	-1902.389974	-1902.750567	-1901.712264	220.31	-1901.816940	-1902.177533
**INT2**	-1902.406063	-1902.766622	-1901.723815	222.82	-1901.829685	-1902.190244
**TS4**	-1902.404809	-1902.765562	-1901.727193	220.71	-1901.832059	-1902.192812
**P1**	-1767.270931	-1767.587428	-1766.689139	203.27	-1766.785719	-1767.102216
**2**	-1575.740001	-1575.928508	-1575.511784	147.93	-1575.582070	-1575.770577
$HN{=}CPh_2$	-556.691518	-556.832108	-556.475550	102.94	-556.524458	-556.665048
**OC3**	-2132.449058	-2132.778097	-2132.002566	205.31	-2132.100114	-2132.429153
**TS5**	-2132.422191	-2132.749282	-2131.977705	200.36	-2132.072902	-2132.399993
**INT3**	-2132.452893	-2132.778924	-2132.006036	203.18	-2132.102573	-2132.428604
**TS6**	-2132.395301	-2132.723139	-2131.953578	199.06	-2132.048157	-2132.375995
**TS7**	-2208.851603	-2209.214029	-2208.384620	206.82	-2208.482885	-2208.845311
**TS8**	-2689.162808	-2689.628561	-2688.502295	254.22	-2688.623082	-2689.088835
Z-**INT4**	-2689.169653	-2689.634128	-2688.504555	257.87	-2688.627079	-2689.091554
E-**INT4**	-2689.165913	-2689.630422	-2688.500849	259.31	-2688.624057	-2689.088566
Z-**TS9**	-2689.165472	-2689.630660	-2688.505029	253.49	-2688.625470	-2689.090658
E-**TS9**	-2689.162615	-2689.627793	-2688.502042	255.29	-2688.623339	-2689.088517
Z-**P2**	-2132.465648	-2132.791046	-2132.018552	198.14	-2132.112696	-2132.438094
E-**P2**	-2132.462599	-2132.787688	-2132.014805	201.91	-2132.110738	-2132.435827
$H_2N{-}N{=}CPh_2$	-612.015102	-612.172149	-611.780775	107.96	-611.832071	-611.989118
$HN{-}N(H){=}CPh_2$	-611.989172	-612.147209	-611.754464	107.42	-611.805501	-611.963538
$H_3N{-}N{=}CPh_2^+$	-612.445755	-612.599304	-612.196154	107.56	-612.247259	-612.400808
$H_2N{-}(H)N{=}CPh_2^+$	-612.453737	-612.608049	-612.204816	107.65	-612.255963	-612.410275
**OC4**	-2187.769148	-2188.115209	-2187.304588	211.18	-2187.404926	-2187.750987
**TS11**	-2187.742756	-2188.087445	-2187.280199	206.48	-2187.378306	-2187.722995
**TS16**	-2187.728950	-2188.072145	-2187.265805	212.15	-2187.366606	-2187.709801
**TS10**	-2187.750085	-2188.093556	-2187.286542	206.41	-2187.384614	-2187.728085
**INT6**	-2187.763680	-2188.106869	-2187.298910	203.06	-2187.395393	-2187.738582
**INT5**	-2187.760815	-2188.103372	-2187.294990	203.21	-2187.391543	-2187.734100
**TS12**	-2187.708194	-2188.052989	-2187.248386	205.39	-2187.345972	-2187.690767
**TS13**	-2264.157424	-2264.536196	-2263.667768	209.97	-2263.767530	-2264.146302

**TS14**	-2799.791781	-2800.291134	-2799.094540	264.93	-2799.220417	-2799.719770
Z-**INT7**	-2799.817790	-2800.317136	-2799.115689	264.81	-2799.241509	-2799.740855
E-**INT7**	-2799.819399	-2800.318290	-2799.117103	264.32	-2799.242689	-2799.741580
Z-**TS15**	-2799.810106	-2800.311158	-2799.113379	266.85	-2799.240169	-2799.741221
E-**TS15**	-2799.817843	-2800.317999	-2799.120398	263.25	-2799.245474	-2799.745630
Z-**P3**	-2187.800309	-2188.143031	-2187.334302	204.47	-2187.431453	-2187.774175
E-**P3**	-2187.796713	-2188.139045	-2187.330485	201.69	-2187.426313	-2187.768645

Table S2. Cartesian atomic coordinates (Å) of the equilibrium structures.

1

C	6.537290	-0.034721	0.587230
C	5.879475	1.338764	0.642254
C	5.991664	-0.860822	-0.571898
C	4.361544	1.228951	0.721435
C	4.474988	-0.989097	-0.496315
C	3.837129	0.399611	-0.452418
H	6.247314	1.912654	1.501698
H	6.153833	1.912361	-0.256640
H	4.065778	0.729843	1.656126
H	3.888582	2.218318	0.715064
H	4.046826	0.928525	-1.393985
H	4.077265	-1.553306	-1.349350
H	4.189586	-1.530093	0.418193
H	6.267344	-0.382494	-1.524518
H	6.442570	-1.860872	-0.583541
H	7.626286	0.068751	0.500245
H	6.347572	-0.566720	1.532979
Pt	-0.701363	-0.086994	-0.059395
P	-2.978897	-0.437412	0.282303
P	-0.567401	-2.462366	-0.060984
C	1.255367	0.162182	-0.264129
C	-2.220491	-3.097579	0.441020
C	-3.328987	-2.201328	-0.096656
H	-2.239245	-3.118591	1.539103
H	-2.344226	-4.132262	0.097635
H	-3.406572	-2.287615	-1.188782
H	-4.305780	-2.479752	0.319013
C	-0.253227	-3.157904	-1.710765
C	0.625868	-3.299152	1.021320
C	-3.450554	-0.201608	2.020071
C	-4.174933	0.530987	-0.673173
H	1.645000	-3.072441	0.689849
H	0.482335	-4.385625	0.995644
H	0.514897	-2.948639	2.052489
H	0.735235	-2.839455	-2.061086
H	-0.997405	-2.789372	-2.424519
H	-0.285358	-4.253671	-1.691457
H	-4.507567	-0.448303	2.174028
H	-3.286081	0.842311	2.308213
H	-2.835639	-0.834375	2.668325
H	-5.185356	0.135452	-0.518081
H	-3.935820	0.491776	-1.740210
H	-4.153203	1.576639	-0.350320
N	-1.020674	4.128190	0.944932
C	-0.807341	2.810283	0.970597
C	-1.462093	4.629941	-0.215186
C	-1.030366	1.965708	-0.132436

C	-1.680299	3.819003	-1.322042
N	-1.473208	2.495621	-1.286308
H	-0.441091	2.396996	1.913265
H	-2.035359	4.238321	-2.264024
H	-1.643679	5.704027	-0.256992
N	2.414249	0.283397	-0.369876

2

N	1.610246	-0.141469	0.117436
C	0.441335	-0.205364	0.162551
Pt	-1.466962	-0.273456	0.241516
Cl	-3.850184	-0.376912	0.347694
Cl	-1.305855	-2.649185	0.375089
C	-1.631917	1.636581	0.135549
N	-1.728766	2.796657	0.071785
C	-1.890860	4.195374	-0.003494
C	3.498060	1.306344	-0.039746
C	4.884754	1.430388	-0.087980
C	2.978244	0.001794	0.066435
C	5.705427	0.306416	-0.034973
C	3.776461	-1.155365	0.123028
C	5.156611	-0.968961	0.068954
C	2.585438	2.487015	-0.106317
H	5.320547	2.424913	-0.170016
H	6.786226	0.426566	-0.075211
H	5.805075	-1.842368	0.109543
C	3.154962	-2.508207	0.235820
H	3.919633	-3.289997	0.268908
H	2.488551	-2.721663	-0.610382
H	2.538547	-2.598374	1.140066
H	1.947606	2.452394	-1.000438
H	3.153211	3.421704	-0.137310
H	1.908128	2.530593	0.757820
H	-1.153375	4.681646	0.641085
H	-2.898941	4.458330	0.328445
H	-1.749606	4.522858	-1.037281

$HNMe_2$

N	-3.982003	2.185858	-0.851846
C	-4.557005	3.483900	-1.150402
H	-4.522234	1.766594	-0.098692
C	-4.055404	1.305943	-2.002938
H	-3.943861	3.989709	-1.908132
H	-4.561462	4.110183	-0.251138
H	-5.591761	3.441668	-1.544891
H	-5.074176	1.195030	-2.424796
H	-3.686459	0.308352	-1.739072
H	-3.415875	1.698682	-2.804637

$HN{=}CPh_2$

H	-1.526324	-2.069234	-1.529754
N	-1.183879	-1.106191	-1.453805
C	-2.180272	-0.308353	-1.271314

C	-2.693975	2.014230	-0.433486
C	-2.371616	3.364302	-0.329049
C	-1.891925	1.145207	-1.185838
C	-1.251472	3.868519	-0.985256
C	-0.760602	1.663647	-1.832598
C	-0.447028	3.013257	-1.738797
H	-3.566309	1.626252	0.090440
H	-2.998249	4.024255	0.268443
H	-1.004826	4.926519	-0.911186
H	0.428097	3.402844	-2.256602
H	-0.137847	0.986882	-2.414357
C	-3.869602	-1.999494	-0.536419
C	-3.588641	-0.773614	-1.155973
C	-5.170523	-2.484983	-0.477979
C	-4.647409	-0.047347	-1.718559
C	-6.212951	-1.759691	-1.052978
C	-5.947242	-0.542265	-1.675964
H	-3.057666	-2.563158	-0.076615
H	-4.444441	0.902226	-2.211815
H	-6.755978	0.026527	-2.131548
H	-7.231626	-2.141646	-1.011973
H	-5.372463	-3.431460	0.020674

$H_2NN=CPh_2$

N	3.939769	-1.453493	0.739560
N	3.320854	-0.252015	0.548333
C	2.038393	-0.211787	0.346016
C	0.050635	1.323082	0.270392
C	-0.505534	2.590879	0.122808
C	1.437877	1.126968	0.176619
C	0.309012	3.692900	-0.120117
C	2.246910	2.250858	-0.068224
C	1.690467	3.513543	-0.212438
H	-0.601336	0.475587	0.475456
H	-1.584304	2.714930	0.205275
H	-0.126359	4.683941	-0.237838
H	2.338857	4.367060	-0.406085
H	3.323096	2.113611	-0.147285
C	1.153235	-2.364675	1.297246
C	1.175812	-1.420434	0.261140
C	0.338567	-3.490583	1.213285
C	0.371846	-1.641503	-0.866375
C	-0.454307	-3.699203	0.086447
C	-0.431116	-2.773932	-0.955796
H	1.774684	-2.204925	2.177642
H	0.380654	-0.913800	-1.677575
H	-1.045574	-2.931850	-1.840535
H	-1.088685	-4.581487	0.020736
H	0.322583	-4.207271	2.032728
H	4.911846	-1.373866	0.461958
H	3.491968	-2.255106	0.289237

$HNNHCPh_2$

N	-1.758005	-2.083546	-1.446312
H	-1.264857	-0.306531	-0.867207
C	-3.088467	-0.029932	-1.622016
C	-4.027669	2.219540	-1.101806
C	-3.877210	3.584112	-0.895264
C	-2.925490	1.411110	-1.443365
C	-2.624903	4.186134	-1.019701
C	-1.672399	2.038983	-1.585812
C	-1.524979	3.403639	-1.365040
H	-5.007592	1.759704	-0.982019
H	-4.745601	4.182979	-0.624739
H	-2.510388	5.256437	-0.858504
H	-0.545309	3.862788	-1.488982
H	-0.809341	1.458510	-1.912383
C	-4.853497	-1.818355	-1.585127
C	-6.050771	-2.345630	-2.056171
C	-4.332485	-0.624574	-2.117880
C	-6.753695	-1.701355	-3.072852
C	-5.060301	0.018597	-3.134617
C	-6.252858	-0.515121	-3.607328
H	-4.328841	-2.321831	-0.773907
H	-6.440113	-3.264156	-1.620234
H	-7.687076	-2.120158	-3.444587
H	-6.793021	-0.002328	-4.401410
H	-4.674207	0.944107	-3.559378
N	-2.036338	-0.804539	-1.313566
H	-2.550149	-2.490690	-1.958405

$H_3NN{=}CPh_2^+$

H	-1.454018	1.555310	0.149298
N	-1.600796	1.705700	-0.861888
H	-0.911257	1.150921	-1.381910
C	-3.804407	1.095413	-0.450029
C	-3.844655	0.225667	1.891815
C	-3.657992	0.407475	3.256914
C	-3.608514	1.285587	1.004314
C	-3.260616	1.649201	3.751430
C	-3.197448	2.529598	1.507160
C	-3.036526	2.710547	2.877353
H	-4.167942	-0.738687	1.503421
H	-3.834605	-0.421127	3.938998
H	-3.130728	1.791788	4.822042
H	-2.741353	3.684341	3.261587
H	-3.049562	3.367896	0.825235
C	-5.196535	0.076839	-2.254373
C	-6.417902	-0.367813	-2.735344
C	-5.093896	0.598162	-0.952567
C	-7.556340	-0.299302	-1.929939
C	-6.244558	0.662213	-0.150098
C	-7.466500	0.215705	-0.639458

H	-4.305983	0.012470	-2.874651
H	-6.485024	-0.777029	-3.741168
H	-8.513648	-0.650756	-2.310064
H	-8.351721	0.275604	-0.010182
H	-6.186995	1.078263	0.853215
N	-2.911109	1.339467	-1.367119
H	-1.411405	2.692232	-1.075882

$H_2NNH{=}CPh_2^+$

H	-0.948224	-2.370769	-1.553832
N	-1.926719	-2.162646	-1.357417
H	-1.253218	-0.347940	-0.809755
C	-3.079604	-0.038812	-1.596733
C	-4.041728	2.186736	-1.073545
C	-3.899366	3.554291	-0.880054
C	-2.928367	1.406622	-1.429248
C	-2.656032	4.160760	-1.049718
C	-1.679861	2.027988	-1.603770
C	-1.548358	3.396083	-1.414970
H	-5.009119	1.711413	-0.924399
H	-4.763395	4.149575	-0.593909
H	-2.550770	5.233932	-0.905141
H	-0.582175	3.870804	-1.569660
H	-0.820865	1.446481	-1.935865
C	-4.847419	-1.813115	-1.561046
C	-6.052225	-2.314476	-2.036102
C	-4.318477	-0.627284	-2.102223
C	-6.725937	-1.661854	-3.067846
C	-5.017282	0.036969	-3.126011
C	-6.204817	-0.489668	-3.614091
H	-4.337026	-2.318401	-0.743582
H	-6.467536	-3.219107	-1.598299
H	-7.662971	-2.066726	-3.444151
H	-6.728862	0.019882	-4.419182
H	-4.611661	0.953505	-3.549004
N	-2.043506	-0.783367	-1.282521
H	-2.492533	-2.504248	-2.135285

OC1

C	-1.186864	0.267390	-0.273325
N	-2.340418	0.460792	-0.268903
N	-0.901571	0.261075	3.211845
C	-6.500142	-0.312710	-0.621318
C	-5.842894	0.625809	-1.626512
C	-5.905559	-0.131558	0.770328
C	-4.330081	0.446480	-1.652820
C	-4.393204	-0.318610	0.760116
C	-3.757521	0.641202	-0.247646
H	-6.247015	0.463453	-2.633223
H	-6.077219	1.668383	-1.361222
H	-4.073532	-0.568731	-1.991817
H	-3.857003	1.151982	-2.346295

H	-3.930238	1.678439	0.075958
H	-3.963131	-0.153167	1.755512
H	-4.140904	-1.348549	0.464793
H	-6.141546	0.878192	1.140182
H	-6.354735	-0.835924	1.481142
H	-7.584298	-0.144261	-0.596687
H	-6.351883	-1.354539	-0.946188
Pt	0.757140	-0.125859	-0.259764
P	3.017651	-0.686645	-0.340465
P	0.407422	-2.471829	-0.153778
C	2.012331	-3.264255	-0.574071
C	3.161533	-2.479891	0.046493
H	2.094586	-3.274198	-1.669284
H	2.016129	-4.309532	-0.240414
H	3.147875	-2.571026	1.141726
H	4.134900	-2.854237	-0.294988
C	-0.019023	-3.092674	1.499680
C	-0.829672	-3.216027	-1.253317
C	3.727034	-0.473187	-1.997470
C	4.177803	0.147839	0.772134
H	-0.800271	-4.310257	-1.194574
H	-0.651943	-2.910199	-2.289426
H	-1.830123	-2.877116	-0.960494
H	-0.954128	-2.630004	1.835792
H	0.767429	-2.829140	2.214523
H	-0.142680	-4.182192	1.488697
H	4.765053	-0.824839	-2.019421
H	3.705732	0.586964	-2.271903
H	3.144683	-1.028980	-2.739492
H	5.173876	-0.299681	0.675466
H	3.845175	0.061944	1.811697
H	4.240682	1.210440	0.519034
N	1.206346	3.976267	-1.596801
C	0.935395	2.672868	-1.493392
C	1.780617	4.541968	-0.527794
C	1.230423	1.901715	-0.353016
C	2.074504	3.806543	0.613572
N	1.810875	2.495871	0.705837
H	0.456405	2.208254	-2.358662
H	2.538801	4.279246	1.480069
H	2.008333	5.606325	-0.587779
C	0.505442	-0.011790	3.450388
H	-1.407561	0.080024	4.076391
C	-1.104096	1.656329	2.859582
H	1.052354	0.060289	2.496673
H	0.629723	-1.028035	3.841923
H	0.983064	0.693073	4.159171
H	-0.705753	2.366772	3.610018
H	-2.172466	1.862195	2.723982
H	-0.594128	1.870051	1.910849

TS1

C	-1.011921	0.767861	0.844370
N	-2.133288	1.212502	0.764362
N	-0.325655	1.665306	2.641569
C	-5.291270	-0.528836	-1.544221
C	-4.071133	-0.011045	-2.297549
C	-5.645429	0.395820	-0.386909
C	-2.878240	0.166818	-1.365560
C	-4.465176	0.567643	0.561088
C	-3.229217	1.068005	-0.185705
H	-3.802937	-0.687449	-3.119758
H	-4.316448	0.957468	-2.761359
H	-2.561511	-0.809901	-0.968419
H	-2.014197	0.581286	-1.904238
H	-3.439803	2.084156	-0.555066
H	-4.705872	1.259849	1.377916
H	-4.220744	-0.401343	1.024589
H	-5.939345	1.380076	-0.784464
H	-6.512255	0.013432	0.166988
H	-6.145007	-0.636310	-2.225774
H	-5.074894	-1.535923	-1.150183
Pt	0.589386	-0.205639	0.097973
P	2.331604	-1.331214	-0.969100
P	-0.092341	-2.379350	0.773576
C	0.952930	-3.589035	-0.141635
C	2.366248	-3.050869	-0.317367
H	0.478824	-3.754945	-1.118569
H	0.954252	-4.552734	0.383207
H	2.891498	-3.014882	0.646888
H	2.960189	-3.690017	-0.983231
C	0.239503	-2.723539	2.530561
C	-1.794190	-2.977196	0.536051
C	2.061787	-1.493928	-2.759654
C	4.035851	-0.733290	-0.810257
H	-1.895800	-4.000054	0.918869
H	-2.057758	-2.973877	-0.526673
H	-2.499024	-2.333075	1.073755
H	-0.407467	-2.103537	3.160969
H	1.281716	-2.490639	2.773074
H	0.046215	-3.777995	2.761318
H	2.830671	-2.129136	-3.215110
H	2.097476	-0.503858	-3.227295
H	1.075482	-1.926600	-2.957018
H	4.732224	-1.462340	-1.241212
H	4.286309	-0.576980	0.243753
H	4.152067	0.219656	-1.335097
N	0.997322	3.698112	-1.740416
C	0.592671	2.487356	-1.353241
C	2.174423	4.107029	-1.249067
C	1.327576	1.648838	-0.491164

C	2.918140	3.307624	-0.390230
N	2.508641	2.088313	-0.012341
H	-0.375258	2.157051	-1.739368
H	3.873061	3.653484	0.008189
H	2.525086	5.094468	-1.549990
C	0.948053	1.170602	3.136566
H	-1.082148	1.392079	3.266994
C	-0.359165	3.101288	2.424648
H	1.734682	1.425498	2.416893
H	0.903731	0.080868	3.235046
H	1.211004	1.606348	4.112291
H	-0.231228	3.668836	3.358012
H	-1.319847	3.372657	1.973011
H	0.448243	3.376186	1.735438

INT1

C	-0.660239	-1.146053	1.188184
N	-1.828890	-1.579595	0.967864
N	-0.219256	-1.644315	2.602475
C	-4.880951	-1.182991	-1.991604
C	-3.651011	-2.016056	-2.336667
C	-4.504314	0.035296	-1.155973
C	-2.880674	-2.420118	-1.083861
C	-3.739600	-0.377235	0.094369
C	-2.496552	-1.189922	-0.265237
H	-3.935983	-2.909269	-2.908137
H	-2.989668	-1.426463	-2.992263
H	-3.501945	-3.079918	-0.457408
H	-1.976581	-2.989815	-1.343874
H	-1.830399	-0.556036	-0.886089
H	-3.448407	0.500030	0.689600
H	-4.382863	-0.996427	0.739843
H	-3.875836	0.710610	-1.759526
H	-5.398484	0.610109	-0.880966
H	-5.403582	-0.876149	-2.907411
H	-5.589073	-1.805516	-1.421577
Pt	0.634790	0.105635	0.153171
P	1.977675	1.574656	-1.081153
P	2.213346	-1.521722	-0.527965
C	3.144050	-0.782559	-1.936152
C	3.486098	0.669781	-1.628363
H	2.505213	-0.852880	-2.827289
H	4.046950	-1.372717	-2.139486
H	4.220930	0.727723	-0.813297
H	3.930367	1.173204	-2.496264
C	3.542831	-1.900678	0.664679
C	1.683591	-3.147914	-1.148855
C	1.230948	2.181968	-2.626821
C	2.583993	3.081012	-0.267154
H	2.541087	-3.728097	-1.509901
H	0.974858	-3.017401	-1.973652

H	1.182444	-3.717410	-0.358403
H	4.318544	-2.517266	0.194301
H	3.157266	-2.436984	1.538128
H	4.000207	-0.970091	1.018425
H	1.973158	2.728193	-3.221276
H	0.397468	2.856706	-2.405733
H	0.844725	1.345222	-3.218756
H	3.261445	3.637279	-0.925687
H	3.113636	2.824488	0.656314
H	1.738326	3.727475	-0.007179
N	-2.361138	3.231243	-0.046945
C	-1.500252	2.246840	-0.310235
C	-2.383554	3.664034	1.221109
C	-0.653310	1.655621	0.651273
C	-1.559530	3.108168	2.190783
N	-0.705071	2.110682	1.920528
H	-1.482233	1.887384	-1.342772
H	-1.587130	3.468173	3.220225
H	-3.076392	4.470638	1.461545
C	0.259295	-3.047084	2.543108
H	-1.082412	-1.643249	3.155538
C	0.758716	-0.747205	3.262023
H	1.127773	-3.086888	1.882538
H	-0.543144	-3.672043	2.147599
H	0.539263	-3.372901	3.547876
H	0.886228	-1.071557	4.297767
H	0.379344	0.277844	3.215048
H	1.709255	-0.815089	2.726510

TS2

C	0.789209	-1.008588	1.151781
N	1.928180	-1.567319	0.884447
N	0.591651	-1.588871	2.522560
C	4.685037	-0.143674	-2.111723
C	3.288243	-0.548825	-2.565943
C	5.173940	-1.045976	-0.985123
C	2.300311	-0.555380	-1.403098
C	4.189245	-1.064166	0.176928
C	2.789900	-1.474551	-0.287718
H	2.924710	0.123957	-3.354286
H	3.327661	-1.555246	-3.011982
H	2.191809	0.465616	-1.003034
H	1.301448	-0.859993	-1.745389
H	2.854485	-2.501562	-0.685708
H	4.528063	-1.743679	0.969906
H	4.121968	-0.059329	0.625339
H	5.297419	-2.071027	-1.368798
H	6.162414	-0.725965	-0.631171
H	5.385760	-0.170160	-2.956459
H	4.662858	0.899929	-1.757369
Pt	-0.533656	0.207989	0.198339

P	-1.873830	1.542115	-1.173298
P	0.417092	2.284656	0.859078
C	-0.130200	3.528521	-0.384283
C	-1.594282	3.310834	-0.747504
H	0.509200	3.403644	-1.269864
H	0.042000	4.542267	-0.000410
H	-2.248453	3.556292	0.099870
H	-1.897708	3.950836	-1.585774
C	-0.267440	2.906788	2.429502
C	2.202939	2.589736	1.036912
C	-1.292636	1.398503	-2.893520
C	-3.671935	1.332266	-1.280486
H	2.382572	3.637520	1.307132
H	2.732555	2.377215	0.101929
H	2.621815	1.955034	1.825302
H	0.171796	2.363232	3.272405
H	-1.351848	2.758320	2.460329
H	-0.048530	3.974551	2.552402
H	-1.876663	2.042326	-3.562327
H	-1.380631	0.360987	-3.235067
H	-0.236916	1.687112	-2.953420
H	-4.104984	2.090015	-1.943835
H	-4.127218	1.419843	-0.289135
H	-3.906841	0.339997	-1.679239
N	-1.762948	-3.630131	-1.439987
C	-1.100968	-2.509844	-1.141440
C	-2.988895	-3.744197	-0.913723
C	-1.619883	-1.479916	-0.332781
C	-3.531238	-2.743057	-0.117340
N	-2.864040	-1.617887	0.170393
H	-0.094134	-2.422333	-1.558521
H	-4.533070	-2.843966	0.302703
H	-3.546019	-4.654367	-1.136996
C	0.570081	-0.569942	3.576447
H	1.768889	-2.073070	2.088056
C	-0.557364	-2.497428	2.616116
H	-0.332814	0.050342	3.496688
H	1.460471	0.060792	3.491331
H	0.578223	-1.071805	4.548390
H	-0.506617	-3.017212	3.577162
H	-0.510341	-3.229745	1.803586
H	-1.501397	-1.942228	2.549185

OC2

C	-0.946208	0.922367	0.417068
N	-1.905062	0.466372	1.112741
N	-1.313612	2.328989	-0.080697
C	-2.977999	-3.087424	3.216957
C	-1.839472	-2.238104	3.771280
C	-2.942297	-3.127800	1.693169
C	-1.855468	-0.833924	3.179541

C	-2.949235	-1.723878	1.101686
C	-1.801319	-0.878651	1.653751
H	-1.893907	-2.186680	4.866912
H	-0.878090	-2.721296	3.531341
H	-2.776791	-0.309981	3.481480
H	-1.014420	-0.237150	3.561272
H	-0.842896	-1.351214	1.353419
H	-2.882883	-1.760304	0.004060
H	-3.898632	-1.216637	1.341650
H	-2.030863	-3.654214	1.365388
H	-3.789016	-3.706255	1.300541
H	-2.933531	-4.104204	3.629437
H	-3.939096	-2.659670	3.544441
Pt	0.872818	0.116208	-0.191560
P	2.886290	-0.886151	-0.849488
P	2.235964	1.306530	1.329042
C	3.823742	0.375900	1.426223
C	4.267073	-0.038502	0.028845
H	3.647348	-0.508728	2.053344
H	4.589617	0.982160	1.926952
H	4.542925	0.844307	-0.564431
H	5.148460	-0.691425	0.062215
C	2.781946	2.969385	0.808131
C	1.731716	1.522521	3.063885
C	3.067581	-2.638767	-0.386544
C	3.354335	-0.862399	-2.605235
H	2.512627	2.032767	3.640390
H	1.542631	0.544173	3.518578
H	0.808991	2.110562	3.121559
H	3.569749	3.344596	1.472907
H	1.950883	3.682172	0.824293
H	3.171111	2.931858	-0.215414
H	4.083489	-2.990187	-0.604175
H	2.360074	-3.252939	-0.953657
H	2.863911	-2.781245	0.680408
H	4.361732	-1.272219	-2.745390
H	3.328238	0.161280	-2.992812
H	2.647968	-1.465260	-3.186757
N	-1.152691	-3.178963	-2.132249
C	-0.420755	-2.400193	-1.334215
C	-1.700850	-2.574986	-3.196184
C	-0.217098	-1.018809	-1.541520
C	-1.506101	-1.222253	-3.439739
N	-0.780344	-0.442540	-2.624778
H	0.027100	-2.888025	-0.463870
H	-1.955311	-0.741106	-4.310574
H	-2.306537	-3.189550	-3.862374
C	-1.202617	3.323189	1.008467
H	-2.351918	2.272597	-0.347865
C	-0.568017	2.749185	-1.283900

H	-0.169160	3.349926	1.367106
H	-1.871652	3.028444	1.820041
H	-1.484282	4.307810	0.623536
H	-1.022586	3.667480	-1.669109
H	-0.625316	1.950886	-2.030459
H	0.477923	2.937043	-1.023212
N	-3.996214	2.190128	-0.863565
C	-4.561916	3.501425	-1.150059
H	-4.514459	1.763455	-0.097704
C	-4.050441	1.298834	-2.015451
H	-3.952389	4.000672	-1.913553
H	-4.549870	4.116298	-0.243829
H	-5.597488	3.453101	-1.526080
H	-5.064548	1.199578	-2.437320
H	-3.695135	0.303319	-1.726419
H	-3.391716	1.683306	-2.804943

TS3

C	-0.942603	1.048320	0.182731
N	-1.922401	0.743137	0.940787
N	-1.226641	2.326256	-0.549085
C	-3.164132	-2.474236	3.460280
C	-2.096358	-1.520369	3.985344
C	-2.980849	-2.740654	1.970200
C	-2.068419	-0.222786	3.185246
C	-2.955604	-1.439145	1.179516
C	-1.868197	-0.495824	1.695927
H	-2.259871	-1.306061	5.049911
H	-1.110750	-2.008840	3.915070
H	-3.019940	0.318260	3.315291
H	-1.275438	0.446856	3.548956
H	-0.884579	-0.995035	1.562040
H	-2.795787	-1.631763	0.108370
H	-3.929092	-0.927440	1.265624
H	-2.030839	-3.277077	1.810250
H	-3.773796	-3.400297	1.592625
H	-3.147846	-3.416525	4.024177
H	-4.158499	-2.029039	3.626425
Pt	0.859069	0.070527	-0.197663
P	2.881506	-1.056438	-0.608255
P	2.190279	1.477947	1.155468
C	3.770349	0.569363	1.443612
C	4.242281	-0.064623	0.140836
H	3.581541	-0.199620	2.205394
H	4.525526	1.251937	1.855082
H	4.518017	0.711347	-0.587332
H	5.129750	-0.691806	0.293884
C	2.781348	3.049817	0.437196
C	1.614345	1.933699	2.821052
C	3.049528	-2.702854	0.154463
C	3.380635	-1.328050	-2.335140

H	2.388139	2.483118	3.370884
H	1.356445	1.029326	3.383034
H	0.717586	2.559863	2.754481
H	3.505573	3.529369	1.107198
H	1.952671	3.744317	0.263834
H	3.266264	2.860210	-0.527098
H	4.072025	-3.082399	0.038269
H	2.361714	-3.410277	-0.320822
H	2.806178	-2.655799	1.221534
H	4.396286	-1.736594	-2.399189
H	3.338879	-0.385707	-2.891453
H	2.689117	-2.033617	-2.809435
N	-1.118803	-3.537818	-1.512390
C	-0.413153	-2.609389	-0.865888
C	-1.645506	-3.157686	-2.685722
C	-0.213177	-1.290511	-1.332205
C	-1.459187	-1.873923	-3.179811
N	-0.759405	-0.940524	-2.516643
H	0.014247	-2.913359	0.093973
H	-1.892134	-1.575600	-4.136551
H	-2.226093	-3.898413	-3.236060
C	-1.150713	3.490163	0.348673
H	-2.451601	2.226109	-0.888061
C	-0.412227	2.523768	-1.755058
H	-0.139233	3.594799	0.763351
H	-1.865807	3.356472	1.164664
H	-1.399248	4.395856	-0.214352
H	-0.817593	3.374244	-2.314938
H	-0.459827	1.619741	-2.370992
H	0.634839	2.731268	-1.497674
N	-3.735202	2.086883	-1.202072
C	-4.288874	3.336279	-1.731807
H	-4.198454	1.854242	-0.322886
C	-3.877920	0.957403	-2.126056
H	-3.728428	3.623736	-2.627205
H	-4.190096	4.124497	-0.980046
H	-5.347083	3.226373	-1.999635
H	-4.926392	0.796911	-2.406631
H	-3.488124	0.053269	-1.648617
H	-3.294652	1.161313	-3.030326

INT2

C	0.855644	-0.655386	-1.060555
N	1.887284	-1.010674	-0.333338
N	0.925049	-0.866238	-2.418392
C	2.797574	-0.814196	3.877314
C	2.236180	-2.107962	3.297283
C	2.183845	0.408066	3.202180
C	2.393949	-2.155997	1.781206
C	2.361139	0.342757	1.690765
C	1.752918	-0.938285	1.116921

H	2.718328	-2.979227	3.761187
H	1.164316	-2.179036	3.547679
H	3.466427	-2.178590	1.519865
H	1.955476	-3.077499	1.371321
H	0.677912	-0.934841	1.393208
H	1.911687	1.218766	1.202622
H	3.436867	0.368900	1.448178
H	1.105104	0.451568	3.433015
H	2.621447	1.332705	3.602801
H	2.636972	-0.778657	4.963342
H	3.889532	-0.795579	3.723460
Pt	-0.947036	0.114700	-0.277107
P	-2.980446	0.739657	0.712748
P	-1.932612	-2.018465	-0.376209
C	-3.582055	-1.950906	0.446988
C	-4.206169	-0.569822	0.283593
H	-3.420400	-2.173112	1.510697
H	-4.237949	-2.737690	0.052544
H	-4.508260	-0.399994	-0.759264
H	-5.106896	-0.463680	0.902340
C	-2.274844	-2.592707	-2.071921
C	-1.099606	-3.447533	0.385314
C	-2.958578	0.709736	2.539011
C	-3.827878	2.300440	0.316793
H	-1.719525	-4.349882	0.316455
H	-0.890316	-3.244321	1.441424
H	-0.148741	-3.638710	-0.124945
H	-2.817867	-3.545611	-2.063307
H	-1.328164	-2.730607	-2.606600
H	-2.870197	-1.850791	-2.615276
H	-3.974393	0.780127	2.947488
H	-2.368207	1.547417	2.926352
H	-2.493038	-0.216202	2.895952
H	-4.797306	2.364568	0.825720
H	-3.986862	2.379157	-0.763777
H	-3.212464	3.150547	0.631685
N	0.357591	4.108964	0.843057
C	-0.237395	2.917442	0.772363
C	1.110550	4.448661	-0.211806
C	-0.097461	2.006553	-0.301197
C	1.249777	3.596809	-1.299252
N	0.674295	2.388586	-1.348288
H	-0.853353	2.648913	1.635607
H	1.857933	3.886865	-2.158791
H	1.609365	5.417869	-0.179632
C	2.062544	-1.504670	-3.040685
H	4.641667	-0.932574	-2.059598
C	0.025807	-0.222957	-3.346731
H	1.729675	-2.033258	-3.942648
H	2.503656	-2.229068	-2.349040

H	2.839422	-0.785648	-3.353330
H	0.511361	0.618537	-3.866138
H	-0.843515	0.169059	-2.806609
H	-0.315434	-0.939884	-4.107165
N	4.472094	-0.457713	-1.169568
C	5.533654	-0.848234	-0.218782
H	3.491934	-0.787917	-0.831199
C	4.389911	0.998870	-1.415060
H	6.504193	-0.539047	-0.613313
H	5.510015	-1.931725	-0.088338
H	5.354065	-0.354149	0.739232
H	4.134513	1.495201	-0.475273
H	3.604635	1.182976	-2.153038
H	5.349848	1.365076	-1.785414

TS4

C	0.865640	-0.646002	-1.065640
N	1.909757	-0.977693	-0.332228
N	0.932991	-0.868775	-2.414245
C	2.818887	-0.686628	3.877277
C	2.290073	-2.003624	3.319111
C	2.176336	0.507668	3.179982
C	2.453835	-2.076398	1.804352
C	2.361166	0.421357	1.670734
C	1.785888	-0.884860	1.120853
H	2.791515	-2.854692	3.799302
H	1.219547	-2.095549	3.568368
H	3.526972	-2.079061	1.547836
H	2.036949	-3.014287	1.409826
H	0.711492	-0.903134	1.390387
H	1.890595	1.275521	1.165199
H	3.436245	0.470584	1.431934
H	1.095993	0.527022	3.406633
H	2.588545	1.450095	3.565294
H	2.654772	-0.636491	4.962101
H	3.910228	-0.643772	3.724980
Pt	-0.939891	0.104387	-0.274202
P	-2.976822	0.717439	0.708950
P	-1.893944	-2.047551	-0.328309
C	-3.539947	-1.987513	0.501134
C	-4.183684	-0.618952	0.311127
H	-3.371720	-2.187213	1.568347
H	-4.185416	-2.791316	0.124321
H	-4.490457	-0.475754	-0.734309
H	-5.084106	-0.512216	0.930097
C	-2.234675	-2.662868	-2.009360
C	-1.031634	-3.445720	0.455678
C	-2.951149	0.732609	2.534388
C	-3.843602	2.257103	0.275531
H	-1.637855	-4.358792	0.410564
H	-0.816141	-3.218264	1.505496

H	-0.082623	-3.632183	-0.059986
H	-2.750426	-3.630132	-1.975519
H	-1.289208	-2.785809	-2.549987
H	-2.855306	-1.949281	-2.562361
H	-3.967525	0.798841	2.941910
H	-2.372806	1.588538	2.899073
H	-2.471552	-0.177159	2.913079
H	-4.813920	2.319038	0.782908
H	-4.003399	2.307884	-0.806542
H	-3.240390	3.122676	0.570961
N	0.306795	4.139909	0.765871
C	-0.273890	2.940014	0.719673
C	1.059388	4.465526	-0.293473
C	-0.120198	2.009471	-0.333989
C	1.212792	3.592159	-1.361936
N	0.650013	2.377366	-1.386757
H	-0.888263	2.682189	1.587076
H	1.821403	3.869878	-2.225088
H	1.546842	5.440892	-0.280545
C	2.051379	-1.535833	-3.044309
H	4.544114	-1.020529	-2.019102
C	0.007721	-0.261535	-3.343800
H	1.689361	-2.086618	-3.921221
H	2.500005	-2.248581	-2.345327
H	2.823831	-0.833049	-3.398071
H	0.478636	0.568726	-3.891948
H	-0.855840	0.136522	-2.799538
H	-0.336686	-1.003871	-4.077324
N	4.376573	-0.503070	-1.154512
C	5.421811	-0.877937	-0.187543
H	3.283992	-0.807435	-0.788376
C	4.350657	0.937657	-1.463307
H	6.409249	-0.607506	-0.574309
H	5.377287	-1.955783	-0.014449
H	5.250674	-0.350435	0.755069
H	4.108140	1.487708	-0.548962
H	3.574737	1.126271	-2.211393
H	5.321839	1.267723	-1.844023

P1

C	-0.739647	-0.702466	1.531313
N	-1.917384	-1.180508	1.111182
N	-0.481765	-0.785570	2.843750
C	-4.472720	-1.261865	-2.347677
C	-3.409621	-2.343125	-2.189103
C	-3.974396	0.084461	-1.833930
C	-2.924527	-2.441272	-0.747140
C	-3.489650	-0.019080	-0.392514
C	-2.409688	-1.090410	-0.261919
H	-3.793127	-3.315392	-2.524376
H	-2.550659	-2.107859	-2.839249

H	-3.756569	-2.758077	-0.096668
H	-2.137940	-3.200976	-0.646773
H	-1.539736	-0.794428	-0.872534
H	-3.101171	0.944368	-0.033487
H	-4.332613	-0.285981	0.266034
H	-3.141411	0.430528	-2.467724
H	-4.760792	0.845944	-1.914168
H	-4.781865	-1.181662	-3.398069
H	-5.370202	-1.554164	-1.779861
Pt	0.629430	0.121292	0.175731
P	2.210437	0.974771	-1.304533
P	1.795894	-1.912196	-0.114646
C	3.109416	-1.633704	-1.379561
C	3.626033	-0.202319	-1.306762
H	2.660336	-1.829330	-2.362970
H	3.919168	-2.362381	-1.248036
H	4.188120	-0.037664	-0.377017
H	4.302892	0.024856	-2.140564
C	2.681002	-2.530181	1.352370
C	0.884721	-3.368897	-0.712423
C	1.702450	1.048974	-3.051967
C	2.936391	2.605665	-0.976149
H	1.571377	-4.195625	-0.932018
H	0.326332	-3.122130	-1.622310
H	0.173850	-3.703522	0.051105
H	3.263390	-3.427669	1.111739
H	1.962211	-2.779324	2.140856
H	3.359186	-1.761941	1.739468
H	2.559744	1.285794	-3.693664
H	0.938261	1.819833	-3.196240
H	1.278417	0.088655	-3.365178
H	3.746946	2.821477	-1.682120
H	3.330435	2.642322	0.044700
H	2.169563	3.381912	-1.074082
N	-1.594832	3.735434	-0.689328
C	-0.883503	2.608698	-0.751559
C	-1.778580	4.242107	0.537025
C	-0.356350	1.935829	0.372033
C	-1.252157	3.620897	1.662050
N	-0.559118	2.475785	1.593824
H	-0.728023	2.196563	-1.752541
H	-1.397346	4.050271	2.654629
H	-2.357646	5.162442	0.615718
C	-1.444535	-1.337180	3.789189
C	0.731840	-0.239937	3.425445
H	-1.015943	-1.301839	4.792162
H	-1.676605	-2.383213	3.551854
H	-2.376807	-0.757432	3.794397
H	0.487620	0.577441	4.115100
H	1.373165	0.152400	2.629950

H	1.271272	-1.017460	3.980331
H	-2.601473	-1.519315	1.783918

OC3

N	-2.423785	-0.649152	-0.182416
C	-1.379076	-0.532714	-0.697444
Pt	0.319242	-0.252453	-1.524068
H	0.781964	-2.088940	1.889784
N	0.396327	-1.139876	1.864692
Cl	2.468690	0.112605	-2.506326
Cl	0.628813	-2.630584	-1.545158
C	0.035536	1.643454	-1.567633
C	1.360497	-0.283529	1.870271
C	1.914567	2.121930	1.365562
C	1.556829	3.466756	1.313172
C	1.008453	1.158600	1.831593
C	0.288586	3.870537	1.723860
C	-0.274676	1.575154	2.219649
C	-0.629318	2.917889	2.170451
H	2.901322	1.812682	1.024152
H	2.274527	4.199945	0.947949
H	0.013775	4.924324	1.697004
H	-1.622046	3.227069	2.495905
H	-0.982558	0.827422	2.574762
C	3.216028	-1.768220	1.117731
C	2.791905	-0.682395	1.895834
C	4.537946	-2.196791	1.164633
C	3.720119	-0.039963	2.726804
C	5.449618	-1.561953	2.006052
C	5.036358	-0.486450	2.790535
H	2.506015	-2.251041	0.443912
H	3.402121	0.802776	3.340001
H	5.743260	0.009622	3.453660
H	6.483491	-1.900727	2.046242
H	4.856843	-3.027034	0.536999
N	-0.183743	2.788028	-1.610198
C	-0.420735	4.174412	-1.700480
C	-4.444537	0.420871	0.482720
C	-5.675555	0.315458	1.127157
C	-3.640552	-0.733825	0.452055
C	-6.078858	-0.885812	1.705189
C	-4.015155	-1.962875	1.023927
C	-5.257540	-2.009174	1.654897
C	-3.978578	1.687161	-0.158792
H	-6.323097	1.189868	1.168979
H	-7.045292	-0.946984	2.201988
H	-5.579951	-2.944537	2.109718
C	-3.107928	-3.145341	0.941305
H	-3.521338	-3.998426	1.487587
H	-2.114407	-2.921465	1.350840
H	-2.945944	-3.456752	-0.099376

H	-3.016223	2.022257	0.253502
H	-4.707359	2.491307	-0.018316
H	-3.822125	1.558331	-1.238867
H	-0.622014	4.440038	-2.742415
H	0.460284	4.718300	-1.347757
H	-1.284876	4.436826	-1.082608

TS5

N	1.744561	-0.025151	-1.421929
C	0.835093	-0.331689	-0.695336
Pt	0.145652	-0.908259	1.033774
H	-1.146589	-1.071522	-2.152151
N	-0.916686	-0.174239	-1.718971
Cl	-0.634753	-1.639273	3.207853
Cl	-0.564837	-2.975377	0.019918
C	0.715114	0.717000	1.856482
C	-1.938642	0.517402	-1.324024
C	-2.470659	2.210574	0.436982
C	-2.169377	3.381891	1.123269
C	-1.671976	1.795647	-0.638629
C	-1.084388	4.163432	0.729257
C	-0.570978	2.578068	-1.015759
C	-0.287748	3.761140	-0.343544
H	-3.314486	1.597348	0.749188
H	-2.786743	3.686388	1.966146
H	-0.860327	5.089329	1.257449
H	0.554851	4.374838	-0.659985
H	0.042287	2.254288	-1.855421
C	-3.633908	-1.307923	-1.492073
C	-3.326831	0.061675	-1.514532
C	-4.942537	-1.733926	-1.680106
C	-4.355876	0.990268	-1.737132
C	-5.956167	-0.804194	-1.910171
C	-5.659423	0.557442	-1.943728
H	-2.847393	-2.034187	-1.281965
H	-4.124097	2.053569	-1.765548
H	-6.447527	1.285213	-2.126330
H	-6.980365	-1.141814	-2.058817
H	-5.174401	-2.796220	-1.637751
N	1.058719	1.711564	2.363915
C	1.439776	2.881886	3.050849
C	3.798982	1.213794	-1.575941
C	5.191472	1.205513	-1.663876
C	3.143110	-0.023654	-1.474105
C	5.902307	0.009490	-1.669189
C	3.832686	-1.249326	-1.466278
C	5.224404	-1.201666	-1.572800
C	3.014367	2.485968	-1.560317
H	5.716224	2.157935	-1.735095
H	6.987757	0.020891	-1.747999
H	5.777777	-2.140418	-1.572741

C	3.095422	-2.542612	-1.343705
H	3.779939	-3.393664	-1.417203
H	2.329517	-2.654318	-2.123061
H	2.565739	-2.622804	-0.384043
H	2.299186	2.530057	-2.393452
H	3.671632	3.358873	-1.632715
H	2.422307	2.583310	-0.639142
H	2.191187	2.632175	3.805433
H	0.563453	3.318030	3.539575
H	1.860764	3.603535	2.344462

INT3

N	-0.937150	-1.910321	0.864465
C	-0.161323	-1.011464	0.388273
Pt	-0.557376	0.577273	-0.799526
H	1.399032	-2.325335	0.731223
N	1.224517	-1.321603	0.683804
Cl	-1.081082	2.488537	-2.265511
Cl	1.211426	-0.136752	-2.293717
C	-1.960537	1.157849	0.331174
C	2.281785	-0.525633	0.725514
C	2.938521	1.834667	0.331891
C	2.752105	3.190487	0.558103
C	2.121612	0.895800	0.985476
C	1.780666	3.621952	1.462042
C	1.146949	1.340443	1.897878
C	0.990137	2.696194	2.143061
H	3.675051	1.493070	-0.392539
H	3.360518	3.915625	0.022665
H	1.641691	4.686741	1.639195
H	0.242940	3.034635	2.858141
H	0.542423	0.610272	2.434404
C	3.750665	-2.265988	-0.256388
C	3.596436	-1.127850	0.557666
C	5.000872	-2.843308	-0.416268
C	4.722925	-0.580256	1.197190
C	6.109746	-2.300708	0.234695
C	5.968989	-1.171182	1.039656
H	2.896982	-2.656463	-0.808577
H	4.608738	0.290465	1.839233
H	6.833633	-0.750067	1.547740
H	7.089366	-2.756999	0.106900
H	5.116491	-3.708547	-1.064934
N	-2.809857	1.580051	1.018540
C	-3.881225	2.012138	1.822627
C	-3.168511	-1.628063	1.721634
C	-4.549351	-1.671110	1.526729
C	-2.322370	-1.903293	0.628719
C	-5.088526	-1.998090	0.285925
C	-2.849408	-2.256450	-0.629387
C	-4.238935	-2.296487	-0.773931

C	-2.584584	-1.252989	3.047393
H	-5.205284	-1.449054	2.369641
H	-6.167935	-2.035018	0.149256
H	-4.652674	-2.571836	-1.744425
C	-1.953835	-2.598157	-1.779630
H	-2.509503	-3.117297	-2.568506
H	-1.117144	-3.239909	-1.472985
H	-1.504339	-1.701738	-2.230624
H	-1.866111	-2.003717	3.403014
H	-3.364792	-1.140948	3.809180
H	-2.031798	-0.302944	2.998073
H	-4.413482	2.824942	1.319966
H	-3.502227	2.370793	2.785165
H	-4.571429	1.177806	1.990767

TS6

N	0.536867	1.860743	-0.680879
C	0.071887	0.641013	-0.602115
Pt	1.009539	-1.073238	-0.309891
H	-0.760011	2.132683	-0.903524
N	-1.313555	0.946064	-0.806368
Cl	2.163996	-3.204775	0.000008
Cl	0.629716	-1.554296	-2.648276
C	1.416167	-0.631805	1.492683
C	-2.394654	0.401988	-0.318187
C	-2.033614	-2.072098	-0.453700
C	-2.041153	-3.327759	0.145136
C	-2.416978	-0.945082	0.282122
C	-2.404628	-3.462491	1.482964
C	-2.823002	-1.089521	1.617256
C	-2.792618	-2.342782	2.218823
H	-1.734598	-1.960088	-1.493726
H	-1.740571	-4.197613	-0.435149
H	-2.390534	-4.443667	1.953950
H	-3.083225	-2.446872	3.262547
H	-3.136214	-0.213834	2.185091
C	-3.574403	2.603821	-0.475364
C	-3.622699	1.205485	-0.332078
C	-4.742265	3.350156	-0.489299
C	-4.874965	0.579541	-0.205384
C	-5.980076	2.716215	-0.370612
C	-6.042870	1.331570	-0.231261
H	-2.613425	3.107761	-0.555536
H	-4.930376	-0.502612	-0.110068
H	-7.005998	0.833316	-0.144222
H	-6.895653	3.304383	-0.384799
H	-4.689933	4.432147	-0.589276
N	1.689643	-0.343387	2.592912
C	2.082273	0.028368	3.893894
C	1.908493	2.937044	0.971798
C	3.151239	3.428472	1.372831

C	1.815673	2.323420	-0.290894
C	4.259221	3.329553	0.536197
C	2.915100	2.216405	-1.156228
C	4.137323	2.731489	-0.713704
C	0.701805	3.044554	1.852099
H	3.241532	3.899186	2.351926
H	5.221761	3.721916	0.859626
H	5.003478	2.657754	-1.370735
C	2.782832	1.557058	-2.491277
H	3.663960	1.745474	-3.113516
H	1.895538	1.905865	-3.034908
H	2.669221	0.468205	-2.393136
H	-0.060152	3.707839	1.417887
H	0.963909	3.446288	2.837089
H	0.209319	2.073008	2.002315
H	2.862114	0.794455	3.835115
H	2.472578	-0.846433	4.421816
H	1.220939	0.425957	4.439473

TS7

N	-0.890668	-1.900036	0.569520
C	-0.095457	-0.937255	0.180592
Pt	-0.600926	0.753653	-0.793246
H	-0.160060	-3.016856	0.899912
O	0.842071	-3.606280	0.973645
H	0.851872	-4.228452	0.224027
H	1.329795	-2.579330	0.598668
N	1.259153	-1.277113	0.373194
Cl	-1.187127	2.827938	-1.982779
Cl	1.091977	0.262421	-2.446263
C	-1.959016	1.178739	0.457430
C	2.253530	-0.443870	0.572601
C	2.791685	1.992151	0.492903
C	2.544806	3.295085	0.905349
C	2.048693	0.931906	1.035606
C	1.592769	3.549365	1.892550
C	1.093622	1.197821	2.031524
C	0.880535	2.497832	2.468503
H	3.521171	1.792502	-0.289836
H	3.093257	4.117320	0.451210
H	1.408930	4.571808	2.217348
H	0.146914	2.692943	3.248987
H	0.539771	0.370648	2.475256
C	3.832489	-2.006305	-0.538048
C	3.608603	-0.959093	0.373931
C	5.111878	-2.502344	-0.735837
C	4.699282	-0.423195	1.080050
C	6.185659	-1.969490	-0.020862
C	5.976884	-0.932765	0.887001
H	2.996122	-2.392255	-1.118280
H	4.535410	0.377641	1.798431

H	6.813252	-0.519996	1.447160
H	7.189177	-2.361371	-0.176444
H	5.277622	-3.297753	-1.459329
N	-2.782914	1.505144	1.223061
C	-3.800574	1.874933	2.121698
C	-3.090171	-1.779686	1.550892
C	-4.477580	-1.817145	1.409947
C	-2.295581	-1.888312	0.394467
C	-5.063293	-1.971352	0.156190
C	-2.865459	-2.074385	-0.877406
C	-4.260167	-2.105599	-0.970526
C	-2.450401	-1.583886	2.890508
H	-5.100660	-1.730306	2.300686
H	-6.147189	-2.002878	0.059824
H	-4.714769	-2.247906	-1.950811
C	-2.019037	-2.250975	-2.099248
H	-2.578936	-2.756062	-2.894111
H	-1.113363	-2.836029	-1.892720
H	-1.676423	-1.287015	-2.501171
H	-1.708602	-2.363319	3.110130
H	-3.197286	-1.592794	3.692157
H	-1.911232	-0.626507	2.949017
H	-4.412394	2.664935	1.676344
H	-3.356863	2.243521	3.052431
H	-4.433499	1.007688	2.339518

TS8

N	-0.797457	-1.084638	1.407094
C	-0.811930	-0.358436	0.354668
Pt	-2.099207	-0.284457	-1.203756
H	2.300426	-1.967503	-0.746018
N	2.357042	-1.242127	-0.030974
H	1.342865	-0.350425	0.039296
N	0.378940	0.402603	0.120690
Cl	-3.643756	-0.169287	-3.148288
Cl	-0.252174	-0.911287	-2.657840
C	-3.565167	0.163620	-0.101920
C	0.450498	1.690611	-0.051545
C	-1.108353	3.575092	-0.544310
C	-2.217050	4.343615	-0.208293
C	-0.668860	2.565679	0.323845
C	-2.864805	4.143290	1.010449
C	-1.321536	2.371723	1.549607
C	-2.404976	3.169586	1.896476
H	-0.603353	3.722903	-1.497755
H	-2.576903	5.102474	-0.899923
H	-3.725705	4.755320	1.274005
H	-2.894577	3.028359	2.859214
H	-0.954744	1.607775	2.233966
C	2.386162	1.545687	-1.607133
C	1.674106	2.256823	-0.626635

C	3.552551	2.075990	-2.143324
C	2.143496	3.511460	-0.207328
C	4.024966	3.312858	-1.703160
C	3.319315	4.028961	-0.736760
H	1.981596	0.601609	-1.972309
H	1.596071	4.065626	0.553965
H	3.687093	4.994199	-0.394502
H	4.940678	3.725289	-2.123419
H	4.087823	1.530749	-2.918726
N	-4.512222	0.456213	0.523261
C	-5.667975	0.749644	1.270007
C	-2.798004	-1.750090	2.616793
C	-3.777252	-2.712353	2.873420
C	-1.817395	-2.027073	1.645568
C	-3.776358	-3.933980	2.207623
C	-1.778099	-3.274720	0.991354
C	-2.772882	-4.209506	1.283706
C	-2.783061	-0.444813	3.349753
H	-4.544451	-2.494592	3.617982
H	-4.545309	-4.675247	2.418473
H	-2.749108	-5.172886	0.773596
C	-0.672867	-3.600723	0.036613
H	-0.735266	-4.643432	-0.294617
H	0.311126	-3.451480	0.503460
H	-0.691782	-2.964378	-0.859081
H	-1.809181	-0.264297	3.827150
H	-3.552438	-0.417170	4.130350
H	-2.956043	0.406269	2.676883
H	-6.530018	0.818289	0.599757
H	-5.543014	1.704482	1.791622
H	-5.846788	-0.043153	2.004510
C	3.543244	-1.014934	0.437900
C	5.810552	-2.030163	0.751227
C	6.908358	-2.717154	0.246901
C	4.733514	-1.705980	-0.087435
C	6.954553	-3.067502	-1.101398
C	4.788198	-2.067234	-1.442591
C	5.895136	-2.737129	-1.946170
H	5.769802	-1.763709	1.806007
H	7.731758	-2.979998	0.907943
H	7.819755	-3.596522	-1.496806
H	5.934237	-2.999481	-3.001200
H	3.968809	-1.792721	-2.107709
C	2.631628	0.194130	2.420534
C	2.727616	1.197842	3.376637
C	3.673300	-0.001301	1.498660
C	3.855060	2.018969	3.420768
C	4.806721	0.822536	1.557580
C	4.892742	1.831423	2.509508
H	1.756546	-0.456134	2.386749

H	1.922183	1.336451	4.095990
H	3.926500	2.804541	4.171066
H	5.769397	2.475411	2.536712
H	5.605946	0.687728	0.830488

***Z*-INT4**

N	0.102438	1.148378	-0.530795
C	0.622568	-0.038825	-0.506713
Pt	2.582844	-0.548909	-0.612120
N	-0.295307	-1.082542	-0.527324
Cl	4.991509	-1.207947	-0.701795
Cl	2.094772	-1.958817	-2.516241
C	3.041038	0.571987	0.835616
C	-0.378788	-2.023997	0.350795
C	1.203789	-3.143843	1.937304
C	2.020905	-3.105283	3.062690
C	0.417099	-2.034756	1.602299
C	2.030851	-1.978657	3.884540
C	0.430268	-0.908413	2.432986
C	1.224722	-0.884535	3.574510
H	1.200254	-4.020847	1.291166
H	2.651772	-3.959662	3.300146
H	2.664263	-1.956853	4.769661
H	1.220787	-0.005928	4.219385
H	-0.194715	-0.053373	2.177816
C	-1.820067	-3.339837	-1.208128
C	-1.344783	-3.110453	0.094266
C	-2.772217	-4.321724	-1.448002
C	-1.847661	-3.892070	1.144386
C	-3.272619	-5.087488	-0.392717
C	-2.809238	-4.868737	0.902426
H	-1.412458	-2.748338	-2.026404
H	-1.493500	-3.721058	2.159862
H	-3.195046	-5.462318	1.729378
H	-4.019242	-5.856967	-0.582115
H	-3.120846	-4.499965	-2.463905
N	3.395103	1.244723	1.728621
C	3.821655	2.084114	2.772828
C	0.927047	3.165441	0.520299
C	1.606296	4.381677	0.424145
C	0.862252	2.330417	-0.612093
C	2.191625	4.781876	-0.773300
C	1.424862	2.735206	-1.840106
C	2.090365	3.962874	-1.894053
C	0.292619	2.741585	1.807712
H	1.664419	5.022550	1.305382
H	2.715698	5.734327	-0.837340
H	2.529900	4.277345	-2.841222
C	1.289225	1.880717	-3.061205
H	1.641953	2.409256	-3.953816
H	0.245312	1.580415	-3.233355

H	1.861279	0.945676	-2.977794
H	-0.731439	2.373970	1.653016
H	0.252906	3.570130	2.525472
H	0.844691	1.915625	2.279348
H	4.272235	1.484919	3.571000
H	2.967515	2.639852	3.176937
H	4.562155	2.796466	2.395095
H	-1.605395	0.917958	-0.948107
N	-2.578216	0.536791	-1.085261
C	-3.618850	1.071099	-0.503179
C	-6.107338	0.985590	-0.491065
C	-7.290566	0.259279	-0.459508
C	-4.871235	0.320656	-0.426532
C	-7.256449	-1.129480	-0.342376
C	-4.847407	-1.080873	-0.310391
C	-6.034337	-1.797191	-0.262791
H	-6.134082	2.067982	-0.600367
H	-8.243727	0.778798	-0.528506
H	-8.186125	-1.694111	-0.308689
H	-6.006185	-2.879129	-0.153328
H	-3.896644	-1.605294	-0.211325
C	-2.547420	3.313845	-0.497296
C	-3.471082	2.411258	0.060171
C	-2.362649	4.565356	0.072864
C	-4.202971	2.789891	1.198745
C	-3.084494	4.926404	1.210851
C	-4.001619	4.038316	1.772083
H	-1.999677	3.042739	-1.398735
H	-4.901320	2.088716	1.650530
H	-4.560020	4.318138	2.662492
H	-2.934738	5.906402	1.659858
H	-1.653256	5.259797	-0.372220
H	-2.635799	-0.377343	-1.526402

E-**INT4**

N	0.285481	1.003935	0.262297
C	-0.786390	0.352875	-0.073583
Pt	-0.688598	-1.678953	-0.157365
N	-1.909926	1.004880	-0.527476
Cl	-0.506861	-4.182284	-0.233737
Cl	-1.291288	-1.592278	-2.491947
C	-0.141883	-1.731167	1.645359
C	-3.139687	0.707046	-0.249638
C	-4.502878	-1.224707	0.580182
C	-4.811507	-2.161505	1.560165
C	-3.535220	-0.237356	0.822018
C	-4.189026	-2.104389	2.806683
C	-2.909612	-0.197456	2.074623
C	-3.243376	-1.113724	3.063930
H	-4.981947	-1.278408	-0.396455
H	-5.541870	-2.940482	1.350221

H	-4.441386	-2.831996	3.576154
H	-2.755419	-1.060236	4.036486
H	-2.156007	0.563862	2.268507
C	-3.889461	1.861414	-2.328338
C	-4.192544	1.358153	-1.052802
C	-4.868628	2.479266	-3.092721
C	-5.499287	1.503408	-0.562491
C	-6.165460	2.617337	-2.594624
C	-6.476206	2.132352	-1.326715
H	-2.876186	1.745806	-2.708260
H	-5.746169	1.136534	0.432270
H	-7.483106	2.244959	-0.928853
H	-6.931884	3.103551	-3.196087
H	-4.623107	2.851302	-4.085895
N	0.236282	-1.739227	2.757527
C	0.577165	-1.826505	4.121059
C	0.901848	2.977458	-1.006275
C	1.137077	4.351422	-1.052178
C	0.378717	2.416158	0.178189
C	0.863720	5.166924	0.043162
C	0.102766	3.227711	1.292458
C	0.350738	4.601136	1.204124
C	1.177604	2.109246	-2.193424
H	1.538711	4.783720	-1.969355
H	1.055813	6.237626	-0.007969
H	0.136300	5.228505	2.070566
C	-0.450435	2.642330	2.555034
H	-0.194336	3.255876	3.427298
H	-0.080121	1.623092	2.728232
H	-1.548032	2.582090	2.526333
H	1.820830	1.254283	-1.942645
H	1.664614	2.674695	-2.995793
H	0.253824	1.676354	-2.602170
H	-0.045603	-2.583401	4.608873
H	0.415421	-0.858737	4.608117
H	1.629692	-2.107888	4.227274
H	1.705072	-0.039045	0.193930
N	2.522262	-0.655162	-0.071574
C	3.746065	-0.196547	-0.097410
C	6.126194	-0.866957	-0.339298
C	7.114807	-1.607408	-0.973217
C	4.795293	-0.942211	-0.784301
C	6.791775	-2.412950	-2.064224
C	4.479080	-1.759396	-1.884258
C	5.475502	-2.483758	-2.521312
H	6.371917	-0.250040	0.522816
H	8.140462	-1.557784	-0.614719
H	7.570556	-2.984728	-2.565009
H	5.226548	-3.099445	-3.382371
H	3.460096	-1.793049	-2.268251

C	3.251526	1.432927	1.705979
C	3.997954	1.079616	0.568653
C	3.426127	2.676007	2.295623
C	4.922757	1.993668	0.036803
C	4.332383	3.585092	1.750023
C	5.076806	3.243651	0.621617
H	2.552602	0.715103	2.134560
H	5.484514	1.734665	-0.858681
H	5.776620	3.957110	0.192649
H	4.454773	4.566151	2.204914
H	2.850758	2.940852	3.180408
H	2.290693	-1.560929	-0.474703

***Z*-TS9**

N	0.132753	-1.061018	-0.483371
C	-0.544553	0.058241	-0.445903
Pt	-2.552521	0.263813	-0.596127
N	0.236384	1.192780	-0.441977
Cl	-5.018674	0.520074	-0.770374
Cl	-2.236681	1.882706	-2.361703
C	-2.874706	-0.983652	0.786712
C	0.104716	2.202295	0.354568
C	-1.753304	3.155530	1.739132
C	-2.662498	3.040253	2.785497
C	-0.782172	2.165363	1.540990
C	-2.586286	1.963129	3.667128
C	-0.713853	1.084070	2.429098
C	-1.602104	0.990858	3.494446
H	-1.822514	3.988849	1.041128
H	-3.434913	3.796020	2.912465
H	-3.294586	1.882678	4.489896
H	-1.534303	0.150543	4.185188
H	0.047695	0.318824	2.277991
C	1.472127	3.574109	-1.213058
C	0.899520	3.408181	0.060021
C	2.269146	4.675323	-1.489867
C	1.144706	4.378050	1.043439
C	2.510374	5.631816	-0.501095
C	1.945363	5.480982	0.762961
H	1.266222	2.828853	-1.979691
H	0.716957	4.260356	2.037429
H	2.129410	6.224101	1.536516
H	3.133246	6.497219	-0.720910
H	2.698418	4.797061	-2.482683
N	-3.143689	-1.719245	1.658782
C	-3.445761	-2.627413	2.688859
C	-0.433787	-3.192455	0.519547
C	-0.983986	-4.469991	0.399885
C	-0.461645	-2.341114	-0.599545
C	-1.525200	-4.904389	-0.806693
C	-0.968921	-2.775192	-1.838237

C	-1.504149	-4.064081	-1.915456
C	0.150472	-2.725813	1.816853
H	-0.974890	-5.130859	1.267859
H	-1.948469	-5.904271	-0.888435
H	-1.904335	-4.408566	-2.869109
C	-0.920495	-1.890196	-3.045177
H	-1.101626	-2.464495	-3.960428
H	0.050178	-1.384893	-3.143683
H	-1.676639	-1.093152	-2.999723
H	1.145484	-2.279561	1.681317
H	0.249984	-3.555193	2.527124
H	-0.471305	-1.951196	2.290527
H	-4.023512	-2.122903	3.470202
H	-2.517612	-3.017812	3.121940
H	-4.031728	-3.460870	2.288395
H	1.461567	-0.846359	-0.733070
N	2.578413	-0.415328	-0.956153
C	3.670598	-0.849095	-0.401276
C	6.146892	-0.529957	-0.388250
C	7.259144	0.301981	-0.345189
C	4.855720	0.011892	-0.293429
C	7.098520	1.677212	-0.185539
C	4.704688	1.399765	-0.134327
C	5.819674	2.223965	-0.074119
H	6.272802	-1.601886	-0.528018
H	8.255362	-0.124960	-0.438411
H	7.971851	2.325133	-0.144812
H	5.689711	3.295505	0.064659
H	3.709040	1.829022	-0.019034
C	2.871609	-3.194214	-0.530070
C	3.668950	-2.227959	0.104929
C	2.803843	-4.487377	-0.030052
C	4.403516	-2.585965	1.246541
C	3.522864	-4.828949	1.115048
C	4.320520	-3.878145	1.751188
H	2.325859	-2.927175	-1.434022
H	5.012070	-1.840161	1.755108
H	4.879761	-4.144282	2.645664
H	3.466759	-5.841902	1.509562
H	2.188309	-5.228934	-0.535843
H	2.599136	0.527874	-1.340162

***E*-TS9**

N	0.357295	0.955877	0.135281
C	-0.765801	0.336265	-0.119077
Pt	-0.754845	-1.692713	-0.206480
N	-1.877583	1.047062	-0.475462
Cl	-0.740292	-4.183506	-0.276744
Cl	-1.547716	-1.572878	-2.478153
C	-0.071675	-1.764595	1.551117
C	-3.106120	0.772639	-0.159842

C	-4.458950	-1.162637	0.673938
C	-4.732402	-2.124534	1.639972
C	-3.476728	-0.186758	0.905195
C	-4.062277	-2.103641	2.862481
C	-2.804156	-0.181864	2.134145
C	-3.103074	-1.123514	3.110506
H	-4.974295	-1.189158	-0.285286
H	-5.472402	-2.896038	1.436963
H	-4.286001	-2.852711	3.619887
H	-2.573924	-1.101359	4.062599
H	-2.041188	0.572226	2.320264
C	-3.896044	2.028479	-2.162695
C	-4.169963	1.470093	-0.902759
C	-4.889744	2.690013	-2.869249
C	-5.459884	1.609063	-0.366730
C	-6.170223	2.818786	-2.327166
C	-6.450703	2.281432	-1.073378
H	-2.895152	1.922288	-2.576976
H	-5.681676	1.203710	0.618770
H	-7.443840	2.388356	-0.641289
H	-6.947539	3.340194	-2.883168
H	-4.669193	3.105832	-3.850726
N	0.374742	-1.774224	2.636573
C	0.841455	-1.831226	3.963438
C	1.026579	2.910560	-1.132855
C	1.309965	4.275732	-1.179408
C	0.500006	2.371800	0.057683
C	1.066760	5.098343	-0.082981
C	0.251394	3.187474	1.173069
C	0.538522	4.552880	1.080500
C	1.247322	2.034470	-2.326392
H	1.718387	4.696050	-2.098811
H	1.295147	6.161725	-0.135199
H	0.349757	5.187889	1.946965
C	-0.301971	2.620760	2.443233
H	-0.023580	3.232925	3.309150
H	0.046853	1.594471	2.618874
H	-1.400776	2.587683	2.426272
H	1.885895	1.169847	-2.099696
H	1.713715	2.590474	-3.146877
H	0.300546	1.617856	-2.698039
H	0.330009	-2.636446	4.500230
H	0.642837	-0.879488	4.468293
H	1.919322	-2.022932	3.972142
H	1.498847	0.150836	0.118005
N	2.434823	-0.578840	-0.071768
C	3.676445	-0.190112	-0.085012
C	6.029686	-0.990462	-0.291885
C	6.989342	-1.776737	-0.917032
C	4.710477	-0.971602	-0.772032

C	6.650065	-2.534341	-2.036707
C	4.378838	-1.738701	-1.901241
C	5.346065	-2.508911	-2.531261
H	6.290076	-0.407304	0.589534
H	8.005404	-1.799735	-0.528746
H	7.405400	-3.143872	-2.529176
H	5.083391	-3.088818	-3.413133
H	3.367693	-1.704483	-2.306551
C	3.254365	1.442079	1.735652
C	3.993399	1.067786	0.602161
C	3.480432	2.667453	2.345378
C	4.964020	1.944522	0.093680
C	4.432744	3.541253	1.822094
C	5.170931	3.179447	0.696479
H	2.512929	0.752521	2.139708
H	5.526606	1.669817	-0.796972
H	5.907945	3.864802	0.283615
H	4.597533	4.508577	2.293142
H	2.909179	2.946882	3.228597
H	2.216650	-1.471763	-0.514171

Z-**P2**

N	0.849792	-1.354532	-1.548975
C	-0.024516	-0.881897	-0.657446
Pt	0.486672	-0.055610	1.100024
N	-1.322400	-1.058728	-0.980834
Cl	1.003299	1.073577	3.215634
Cl	-1.087203	-1.486530	2.224874
C	1.749214	1.110313	0.292200
C	-2.253982	-0.154339	-0.890424
C	-2.388311	2.077132	0.244387
C	-1.916948	3.376496	0.401631
C	-1.897586	1.278165	-0.796715
C	-0.991454	3.903719	-0.498525
C	-0.965536	1.814148	-1.696153
C	-0.526896	3.125109	-1.557678
H	-3.094834	1.656329	0.958464
H	-2.269746	3.978483	1.236535
H	-0.634289	4.924604	-0.374778
H	0.188619	3.535666	-2.269398
H	-0.600606	1.192619	-2.514331
C	-3.987199	-1.927764	-0.702518
C	-3.654183	-0.577367	-0.910976
C	-5.312822	-2.331930	-0.718848
C	-4.682359	0.350912	-1.143536
C	-6.327320	-1.398931	-0.946614
C	-6.009593	-0.059452	-1.159980
H	-3.188816	-2.642653	-0.513367
H	-4.436304	1.395098	-1.326771
H	-6.797956	0.667810	-1.342600
H	-7.367490	-1.719437	-0.957971

H	-5.562186	-3.376963	-0.545596
N	2.520177	1.867019	-0.157533
C	3.430223	2.804164	-0.680240
C	2.996022	-0.544248	-2.319920
C	4.385808	-0.519598	-2.211574
C	2.275903	-1.365861	-1.435262
C	5.036448	-1.291983	-1.253556
C	2.907750	-2.182814	-0.485828
C	4.303770	-2.117287	-0.409553
C	2.282066	0.326334	-3.306182
H	4.957419	0.122901	-2.881322
H	6.121658	-1.260117	-1.174006
H	4.814129	-2.738884	0.325422
C	2.146745	-3.095941	0.422232
H	2.758323	-3.960730	0.702789
H	1.221312	-3.462156	-0.036341
H	1.854710	-2.588729	1.352547
H	1.632874	-0.247771	-3.981484
H	2.990830	0.881946	-3.929244
H	1.633150	1.054704	-2.797818
H	3.134524	3.812105	-0.371331
H	3.433295	2.747905	-1.774226
H	4.436384	2.590020	-0.306804
H	0.456525	-1.683735	-2.431031

E-**P2**

N	0.847501	-1.964813	-0.043798
C	0.352751	-0.726563	-0.160716
Pt	-1.537894	-0.495633	-0.805550
N	1.213700	0.277562	0.039176
Cl	-3.858441	-0.166034	-1.545593
Cl	-0.703352	0.817731	-2.633924
C	-2.185200	-1.517183	0.654191
C	0.935033	1.492068	0.435177
C	-1.035793	2.955459	0.877000
C	-2.241691	3.202459	1.521268
C	-0.282182	1.814153	1.199470
C	-2.694651	2.339435	2.517973
C	-0.757779	0.944129	2.191942
C	-1.946055	1.213243	2.857175
H	-0.697103	3.615515	0.080452
H	-2.833264	4.071058	1.240405
H	-3.633660	2.543389	3.029426
H	-2.296497	0.534809	3.633629
H	-0.175976	0.059127	2.447265
C	2.832246	2.342365	-0.926630
C	1.902896	2.543346	0.110823
C	3.752686	3.328183	-1.244599
C	1.941268	3.749228	0.832242
C	3.774710	4.525231	-0.525264
C	2.871515	4.730533	0.515255

H	2.805430	1.407360	-1.483067
H	1.252905	3.908088	1.659529
H	2.894942	5.656525	1.085970
H	4.500726	5.296814	-0.775473
H	4.457532	3.168638	-2.058041
N	-2.562806	-2.117951	1.586785
C	-3.084925	-2.735622	2.741095
C	3.000183	-2.750972	-0.832007
C	4.317621	-3.123176	-0.563188
C	2.212457	-2.303364	0.241919
C	4.829552	-3.048371	0.729049
C	2.704115	-2.214494	1.550514
C	4.030470	-2.597214	1.772738
C	2.442899	-2.794983	-2.219702
H	4.945442	-3.468065	-1.384024
H	5.858882	-3.344838	0.923298
H	4.431123	-2.541276	2.784488
C	1.854048	-1.723284	2.678694
H	2.202791	-2.119909	3.638340
H	0.800526	-2.005442	2.553738
H	1.889631	-0.627227	2.754620
H	1.554062	-3.436420	-2.294293
H	3.183634	-3.174194	-2.930760
H	2.129167	-1.797828	-2.558732
H	-3.982529	-2.200200	3.065656
H	-2.336618	-2.710809	3.540264
H	-3.343493	-3.776009	2.523393
H	0.252730	-2.709860	-0.393037

OC4

C	-2.498486	-0.271545	3.755725
C	-3.317826	-1.314536	3.330427
C	-2.935673	-2.128070	2.265719
C	-1.724157	-1.924531	1.606753
C	-0.929382	-0.853446	2.054068
C	-1.283679	-0.010905	3.123039
C	-1.279992	-2.781282	0.467644
N	0.248093	-0.592039	1.389972
C	1.221088	-0.323916	0.796271
Pt	2.810938	0.130958	-0.168339
C	3.538431	-1.639330	-0.048949
N	3.994308	-2.710644	0.012307
C	4.580445	-3.992161	0.071486
C	-0.395933	1.121726	3.518741
Cl	4.764531	0.742804	-1.399218
Cl	1.950935	2.360054	-0.342611
N	-0.222265	0.150358	-1.876019
N	-1.478540	-0.302973	-2.126153
C	-2.503336	0.248653	-1.543114
C	-3.802515	-0.429414	-1.717495
C	-3.868622	-1.763339	-2.158462

C	-5.090485	-2.399318	-2.330638
C	-6.282801	-1.725545	-2.059986
C	-6.234005	-0.405865	-1.619837
C	-5.010327	0.234796	-1.448482
C	-2.429714	1.481468	-0.712980
C	-2.885521	1.477417	0.612402
C	-2.812087	2.626529	1.394128
C	-2.288730	3.803710	0.862621
C	-1.845046	3.825522	-0.457700
C	-1.912635	2.674823	-1.238270
H	-0.111722	1.106002	-1.530159
H	-3.302078	0.561351	1.033505
H	-3.165202	2.599432	2.424842
H	-2.232592	4.703198	1.473181
H	-1.440335	4.742048	-0.883723
H	-1.575955	2.703895	-2.274907
H	-2.940484	-2.292271	-2.365779
H	-4.991763	1.270227	-1.112669
H	-7.155629	0.134851	-1.409053
H	-7.240194	-2.227789	-2.189320
H	-5.114301	-3.434061	-2.670097
H	-3.583299	-2.937629	1.932248
H	-4.267141	-1.493250	3.832108
H	-2.805006	0.363746	4.585838
H	-0.858186	1.732218	4.300300
H	-0.171557	1.773051	2.662705
H	0.571195	0.764764	3.897954
H	-1.139665	-2.191732	-0.448640
H	-2.012527	-3.567135	0.259309
H	-0.316286	-3.264838	0.679238
H	3.886390	-4.729044	-0.342033
H	4.803718	-4.241784	1.112492
H	5.505359	-3.991772	-0.511296
H	0.395966	-0.048368	-2.653947

TS10

N	0.315662	0.737934	-0.114936
C	-0.554658	-0.055529	-0.374463
Pt	-2.460209	-0.486085	-0.449036
H	0.271267	-2.317193	-0.539482
N	0.428174	-1.625674	-1.277542
Cl	-4.846854	-0.874126	-0.513591
Cl	-2.081549	-2.792159	0.117613
C	-2.771663	1.367039	-0.777653
C	2.597541	-1.223007	-0.659662
C	2.563797	-0.594049	1.760268
C	2.287785	-0.875316	3.093242
C	2.337678	-1.556967	0.766093
C	1.790398	-2.126914	3.455887
C	1.853448	-2.816323	1.142748
C	1.580055	-3.096977	2.479376

H	2.943391	0.387330	1.475214
H	2.450766	-0.109001	3.849717
H	1.569586	-2.346345	4.498761
H	1.202019	-4.079440	2.755787
H	1.712855	-3.593105	0.390282
C	4.149398	-0.203055	-2.337094
C	3.942661	-0.770749	-1.066547
C	5.411324	0.217232	-2.729628
C	5.045577	-0.906732	-0.209578
C	6.498973	0.081515	-1.865065
C	6.310807	-0.484707	-0.607446
H	3.299232	-0.094638	-3.006803
H	4.917191	-1.358165	0.771976
H	7.153143	-0.602359	0.071928
H	7.488334	0.414815	-2.173682
H	5.549166	0.659646	-3.714704
N	-2.891238	2.520238	-0.916230
C	-3.007437	3.919226	-1.042989
C	1.373955	2.882357	0.022340
C	1.635960	4.040027	0.754318
C	0.556210	1.901950	0.613018
C	1.123391	4.205071	2.038256
C	0.021818	2.041340	1.910061
C	0.331772	3.211720	2.606838
C	1.906532	2.678525	-1.358350
H	2.259892	4.813693	0.308009
H	1.348563	5.108698	2.601975
H	-0.062771	3.337866	3.614676
C	-0.835754	0.980182	2.520884
H	-0.943070	1.136072	3.599706
H	-0.427842	-0.028022	2.362617
H	-1.846911	0.968895	2.087613
H	2.414628	1.709356	-1.453121
H	2.615115	3.466272	-1.634480
H	1.101268	2.672574	-2.106809
H	-3.961033	4.246848	-0.619982
H	-2.962979	4.197915	-2.099577
H	-2.183915	4.397530	-0.502434
H	-0.111920	-1.865273	-2.105724
N	1.716794	-1.315016	-1.617598

INT5

N	0.430660	0.813737	0.006349
C	-0.220373	-0.116136	-0.532761
Pt	-2.131069	-0.705653	-0.580410
N	0.695991	-1.067037	-1.371356
Cl	-4.477483	-1.404427	-0.605088
Cl	-1.410871	-3.002832	-0.318266
C	-2.713127	1.093574	-0.758262
C	2.875303	-0.647974	-0.702209
C	2.862018	-0.496930	1.788769

C	2.665957	-1.062902	3.041851
C	2.654268	-1.258799	0.629944
C	2.270352	-2.396221	3.155355
C	2.267239	-2.598838	0.751146
C	2.074391	-3.162644	2.010290
H	3.157957	0.547378	1.696334
H	2.819694	-0.460318	3.935129
H	2.117820	-2.837850	4.138154
H	1.774154	-4.205339	2.090548
H	2.154807	-3.217240	-0.140189
C	4.312864	0.912755	-2.025494
C	4.181097	-0.024566	-0.987011
C	5.543303	1.495575	-2.291979
C	5.313340	-0.363849	-0.229979
C	6.663085	1.150578	-1.533267
C	6.545333	0.218382	-0.505563
H	3.433877	1.185104	-2.605227
H	5.230374	-1.097208	0.569650
H	7.415842	-0.057542	0.085963
H	7.625867	1.614009	-1.741353
H	5.631320	2.230497	-3.089808
N	-3.093202	2.193140	-0.873102
C	-3.542595	3.524541	-0.969145
C	-0.036042	3.113232	0.482949
C	-0.537135	4.094851	1.335357
C	-0.144261	1.761451	0.870688
C	-1.113007	3.753375	2.557394
C	-0.715959	1.393484	2.104860
C	-1.187849	2.417943	2.933540
C	0.573987	3.460581	-0.837659
H	-0.464994	5.140420	1.033991
H	-1.490454	4.529425	3.221179
H	-1.624255	2.146694	3.895183
C	-0.821603	-0.032925	2.552195
H	-0.692096	-0.111968	3.638668
H	-0.079660	-0.680942	2.073271
H	-1.806547	-0.458737	2.313803
H	1.564464	3.000069	-0.956258
H	0.682357	4.544415	-0.955004
H	-0.031249	3.093029	-1.679376
H	-4.583090	3.582287	-0.637205
H	-3.473763	3.863185	-2.007375
H	-2.920971	4.163030	-0.332732
N	2.023115	-0.615717	-1.684399
H	0.637316	-1.985328	-0.894807
H	0.227192	-1.202817	-2.272061

TS11

N	-1.530245	-1.498660	0.638284
C	-0.744076	-0.740788	0.119477
Pt	-0.385024	0.796878	-1.018782

H	0.128761	-3.176857	0.685243
N	1.036187	-2.818079	0.385106
Cl	-0.129129	2.711860	-2.476184
Cl	0.987793	-0.526617	-2.467390
C	-1.469310	1.914979	0.089304
C	2.166206	-0.806912	0.875905
C	4.587820	-0.897666	1.466281
C	5.879571	-1.361397	1.247973
C	3.527825	-1.326413	0.651208
C	6.138027	-2.244028	0.200550
C	3.799494	-2.217318	-0.400623
C	5.096825	-2.661336	-0.626809
H	4.391170	-0.198613	2.278165
H	6.689131	-1.028208	1.894931
H	7.151196	-2.601936	0.025876
H	5.296230	-3.335292	-1.457934
H	2.991967	-2.531172	-1.058936
C	0.988844	0.855708	2.307317
C	1.963038	0.581275	1.335297
C	0.743890	2.161875	2.714568
C	2.703412	1.642227	0.796070
C	1.467457	3.213481	2.153788
C	2.447713	2.949287	1.197365
H	0.432109	0.026051	2.742448
H	3.453432	1.441429	0.032105
H	3.009084	3.768056	0.751341
H	1.275366	4.238564	2.467480
H	-0.013252	2.357522	3.473921
N	-2.118729	2.618778	0.758117
C	-2.829727	3.530962	1.563137
C	-3.649496	-1.529239	1.771451
C	-5.015290	-1.813967	1.738469
C	-2.911210	-1.752422	0.598902
C	-5.612582	-2.324437	0.589676
C	-3.483074	-2.260815	-0.579322
C	-4.849957	-2.546789	-0.552780
C	-2.978501	-0.992036	2.995140
H	-5.609033	-1.636042	2.634718
H	-6.676848	-2.552182	0.584927
H	-5.315533	-2.946608	-1.453190
C	-2.653636	-2.474033	-1.804232
H	-3.218549	-3.005406	-2.576848
H	-1.741013	-3.047967	-1.592010
H	-2.317455	-1.521165	-2.237023
H	-2.166296	-1.650578	3.332827
H	-3.688893	-0.882926	3.821194
H	-2.518373	-0.009293	2.815903
H	-3.708699	3.891160	1.020954
H	-2.181238	4.377654	1.809687
H	-3.150072	3.035586	2.484603

H	1.789514	-3.356494	0.815662
N	1.077396	-1.496618	0.721497

INT6

N	1.001806	-1.633537	-1.211443
C	0.208301	-0.933432	-0.503602
Pt	0.521125	0.338363	1.025456
H	-0.598288	-3.011373	-1.327667
N	-1.445796	-2.629564	-0.900003
Cl	0.905938	1.879779	2.896647
Cl	-1.153622	-0.840536	2.301283
C	1.876687	1.261764	0.072300
C	-2.194817	-0.366254	-0.840623
C	-4.570946	-0.044812	-1.455332
C	-5.917614	-0.352397	-1.314899
C	-3.600104	-0.759642	-0.732309
C	-6.314919	-1.353329	-0.430564
C	-4.010260	-1.771693	0.155289
C	-5.360355	-2.051786	0.309695
H	-4.259218	0.745750	-2.135770
H	-6.659870	0.196746	-1.890324
H	-7.371454	-1.587142	-0.312782
H	-5.671404	-2.818033	1.016561
H	-3.265846	-2.302802	0.744252
C	-0.879828	1.436982	-1.948425
C	-1.847319	1.046778	-1.007328
C	-0.550731	2.777180	-2.097372
C	-2.499309	2.026026	-0.240956
C	-1.172131	3.739399	-1.301924
C	-2.147523	3.361977	-0.378776
H	-0.401850	0.679712	-2.569221
H	-3.246779	1.725066	0.491132
H	-2.631710	4.112839	0.241649
H	-0.904486	4.788977	-1.408858
H	0.193781	3.072325	-2.835297
N	2.703286	1.875223	-0.485302
C	3.717673	2.567275	-1.172868
C	3.197758	-1.034493	-2.002712
C	4.582593	-1.048831	-1.835515
C	2.394190	-1.615319	-1.003081
C	5.162547	-1.637772	-0.715239
C	2.960195	-2.233174	0.128484
C	4.352698	-2.229924	0.247672
C	2.563918	-0.379559	-3.190196
H	5.209394	-0.593778	-2.603639
H	6.245032	-1.648016	-0.600400
H	4.801079	-2.707272	1.119272
C	2.106216	-2.886529	1.170505
H	2.685655	-3.609091	1.756186
H	1.245492	-3.409254	0.733182
H	1.689899	-2.152579	1.875550

H	1.864419	-1.054855	-3.701467
H	3.318102	-0.057713	-3.917375
H	1.978718	0.508303	-2.905768
H	4.228199	3.248621	-0.486077
H	3.280118	3.144636	-1.994173
H	4.441280	1.850454	-1.576799
H	-2.238553	-2.800099	-1.519104
N	-1.217916	-1.253764	-0.834218

TS12

N	1.222037	1.439927	1.118589
C	0.542797	0.533111	0.479811
Pt	1.016617	-0.826404	-0.864914
H	0.089517	1.528337	1.811006
N	-0.740490	0.653211	1.237823
Cl	1.652771	-2.580673	-2.438801
Cl	-0.920441	-0.346499	-2.183057
C	2.639413	-1.203642	0.065521
C	-2.967214	0.598731	0.625969
C	-2.430383	-1.834007	0.902329
C	-2.632635	-3.062622	1.524262
C	-3.179434	-0.712556	1.290450
C	-3.575313	-3.184297	2.542756
C	-4.136778	-0.848282	2.305075
C	-4.326575	-2.075698	2.931040
H	-1.711826	-1.741177	0.085028
H	-2.054866	-3.927343	1.203682
H	-3.731225	-4.145413	3.029288
H	-5.069360	-2.168928	3.721064
H	-4.730096	0.015354	2.602553
C	-4.052419	2.651270	-0.305028
C	-4.114915	1.281497	0.000782
C	-5.124448	3.285355	-0.916097
C	-5.278674	0.568627	-0.324815
C	-6.277594	2.566884	-1.235136
C	-6.349449	1.208036	-0.940652
H	-3.154506	3.208830	-0.049209
H	-5.338082	-0.497204	-0.113167
H	-7.241429	0.638636	-1.194824
H	-7.117668	3.067518	-1.713374
H	-5.064563	4.348485	-1.142208
N	3.653602	-1.427975	0.601555
C	4.890172	-1.674298	1.230486
C	3.440012	1.658380	2.015745
C	4.780979	1.997737	1.843856
C	2.572070	1.805199	0.917383
C	5.242139	2.485148	0.623404
C	3.006051	2.318789	-0.318456
C	4.359415	2.650583	-0.437339
C	2.936083	1.109832	3.313992
H	5.468117	1.874419	2.680980

H	6.291297	2.750348	0.504168
H	4.715085	3.051844	-1.385808
C	2.071897	2.526362	-1.468703
H	2.470392	3.277049	-2.159797
H	1.076591	2.852997	-1.143215
H	1.919969	1.602529	-2.044684
H	2.129998	1.727377	3.732577
H	3.737045	1.059984	4.059456
H	2.519126	0.099043	3.201243
H	4.870129	-1.268778	2.247502
H	5.691600	-1.191995	0.662980
H	5.071895	-2.752268	1.271036
N	-1.825564	1.211165	0.532833
H	-0.961127	-0.223322	1.733159

TS13

N	1.191923	2.275982	-0.158126
C	0.425851	1.249506	0.026399
Pt	0.686614	-0.646484	-0.616189
H	-0.056923	2.984800	-1.616790
O	-0.997207	2.811582	-1.926278
H	-1.438186	2.605539	-1.033678
N	-0.876556	1.574875	0.465397
Cl	0.903821	-2.934526	-1.518350
Cl	-0.961569	-0.114519	-2.389485
C	2.011332	-1.020876	0.667541
C	-2.916367	0.515018	0.593510
C	-3.457977	2.969014	0.363262
C	-4.029524	4.019478	-0.350882
C	-3.576503	1.648892	-0.096827
C	-4.709544	3.765849	-1.539944
C	-4.289674	1.402677	-1.279755
C	-4.839062	2.456812	-2.001585
H	-2.944613	3.168502	1.303125
H	-3.941221	5.037635	0.023317
H	-5.146542	4.588080	-2.103397
H	-5.374377	2.253782	-2.927150
H	-4.388065	0.379746	-1.640488
C	-3.081017	-1.948117	0.960551
C	-3.705454	-0.691514	0.905997
C	-3.821920	-3.084567	1.255484
C	-5.084232	-0.603724	1.142807
C	-5.191138	-2.985561	1.506035
C	-5.819879	-1.743867	1.449389
H	-2.015430	-2.019312	0.746724
H	-5.578857	0.365757	1.099107
H	-6.888086	-1.661947	1.642194
H	-5.768781	-3.878796	1.737707
H	-3.330794	-4.055700	1.279859
N	2.846192	-1.292261	1.441339
C	3.864341	-1.588126	2.367486

C	3.444320	2.044574	0.677470
C	4.814618	1.916217	0.441492
C	2.569550	2.099025	-0.426403
C	5.318119	1.849436	-0.853803
C	3.066086	2.055039	-1.741521
C	4.444039	1.923911	-1.932728
C	2.909835	2.128875	2.074330
H	5.491933	1.873569	1.295460
H	6.388850	1.747320	-1.022082
H	4.827544	1.876859	-2.952124
C	2.139537	2.143040	-2.912869
H	2.681016	2.026954	-3.857768
H	1.628285	3.117634	-2.963514
H	1.369739	1.358155	-2.886335
H	2.362969	3.067461	2.240571
H	3.719635	2.077884	2.811556
H	2.199196	1.322571	2.302103
H	4.789468	-1.083930	2.070972
H	4.035456	-2.668295	2.391736
H	3.566548	-1.246245	3.363862
H	-0.959078	1.856100	-2.324115
N	-1.654369	0.455822	0.887395
H	-0.860323	2.310582	1.172434

TS14

N	1.266565	1.275360	-0.751628
C	1.265042	0.107304	-0.263973
Pt	2.702911	-1.282044	-0.034263
H	-1.059847	-2.414211	-1.677340
N	-2.026754	-2.114941	-1.538946
H	-0.946196	-0.393267	-0.635609
N	-0.078326	-0.308924	0.234546
Cl	4.454554	-3.007672	0.246007
Cl	1.177847	-2.938536	-0.948745
C	3.947245	-0.022217	0.638016
C	-1.420905	0.174381	2.069521
C	-2.605103	0.894668	4.158800
C	-3.215276	1.880646	4.927823
C	-2.080251	1.205313	2.896473
C	-3.321555	3.182500	4.443064
C	-2.194626	2.519661	2.416180
C	-2.812753	3.497230	3.182407
H	-2.521840	-0.120325	4.544370
H	-3.609586	1.629207	5.910662
H	-3.808250	3.949786	5.042829
H	-2.908287	4.508435	2.789075
H	-1.814730	2.750749	1.422577
C	-0.798822	-2.228709	2.496304
C	-1.779097	-1.251914	2.268276
C	-1.157201	-3.567739	2.619203
C	-3.124316	-1.637696	2.193578

C	-2.494205	-3.944856	2.519045
C	-3.476909	-2.977582	2.313608
H	0.248439	-1.937933	2.597560
H	-3.891603	-0.880217	2.031785
H	-4.524257	-3.267386	2.247034
H	-2.771814	-4.993378	2.608710
H	-0.385753	-4.315485	2.790762
N	4.771363	0.698654	1.050715
C	5.778841	1.577960	1.491497
C	2.865621	3.011699	-0.374118
C	3.974431	3.745917	-0.792961
C	2.420031	1.945182	-1.181203
C	4.623923	3.444394	-1.987519
C	3.053229	1.632047	-2.398204
C	4.159449	2.398534	-2.778694
C	2.186431	3.295337	0.928810
H	4.327841	4.566783	-0.167332
H	5.484841	4.030057	-2.305734
H	4.657935	2.163511	-3.719705
C	2.554538	0.513317	-3.259370
H	2.993917	0.560702	-4.262055
H	1.460998	0.532743	-3.365181
H	2.804696	-0.470488	-2.836099
H	1.097017	3.367153	0.816146
H	2.554406	4.225897	1.376116
H	2.352146	2.487538	1.657450
H	6.433196	1.060380	2.199295
H	5.325634	2.443502	1.985742
H	6.367965	1.918478	0.634358
C	-3.105572	-0.029889	-1.650819
C	-4.136059	2.164903	-1.050387
C	-4.036721	3.531336	-0.814083
C	-3.004603	1.422259	-1.426590
C	-2.810360	4.180954	-0.951735
C	-1.779186	2.088529	-1.568397
C	-1.681817	3.453318	-1.327551
H	-5.094711	1.662288	-0.931547
H	-4.921431	4.091428	-0.516605
H	-2.736323	5.252124	-0.770593
H	-0.719008	3.948476	-1.447462
H	-0.895800	1.537531	-1.881503
C	-4.936575	-1.760497	-1.605616
C	-6.142501	-2.255724	-2.091653
C	-4.371425	-0.601213	-2.160058
C	-6.790476	-1.617133	-3.146856
C	-5.043385	0.044218	-3.209965
C	-6.236377	-0.467186	-3.706642
H	-4.437284	-2.267292	-0.782010
H	-6.575851	-3.148349	-1.644069
H	-7.728234	-2.013974	-3.531291

H	-6.737900	0.037015	-4.530354
H	-4.616606	0.948271	-3.642038
H	-0.011011	-1.263967	0.606935
N	-0.597134	0.635522	1.179031
N	-2.046896	-0.749541	-1.402444
H	-2.628499	-2.449203	-2.291990

***Z*-INT7**

N	0.805561	1.109272	0.322360
C	1.252459	-0.107926	0.359789
Pt	3.026278	-0.822315	-0.304288
H	-0.367201	-0.610354	-1.814504
N	-1.377647	-0.479928	-1.699287
H	-0.794892	1.022655	-0.458995
N	0.401472	-1.103256	0.792536
Cl	5.147066	-1.733234	-1.215195
Cl	1.710400	-2.093668	-1.915135
C	4.024823	0.214100	0.916366
C	-1.691366	-1.811023	1.370565
C	-4.050885	-2.425893	1.952180
C	-5.345746	-2.070740	2.320542
C	-3.047603	-1.452581	1.819888
C	-5.664146	-0.740909	2.579102
C	-3.381906	-0.112281	2.092918
C	-4.670490	0.234924	2.470914
H	-3.817666	-3.472362	1.765397
H	-6.107395	-2.843228	2.413741
H	-6.675891	-0.464255	2.870550
H	-4.903855	1.277140	2.685999
H	-2.608810	0.649326	2.004943
C	-0.343660	-3.902150	1.678177
C	-1.356000	-3.211435	0.997876
C	0.036773	-5.178854	1.274677
C	-1.982765	-3.831307	-0.091129
C	-0.586755	-5.781285	0.184819
C	-1.598985	-5.106097	-0.494825
H	0.149574	-3.428567	2.527292
H	-2.768771	-3.302034	-0.630830
H	-2.088218	-5.572561	-1.348227
H	-0.282607	-6.775675	-0.136763
H	0.826994	-5.700777	1.810889
N	4.688785	0.847036	1.645124
C	5.495328	1.666118	2.458243
C	2.088602	2.961889	1.180113
C	2.878712	4.090990	0.956475
C	1.659888	2.201907	0.073466
C	3.230373	4.475091	-0.334863
C	1.988027	2.591776	-1.239524
C	2.780004	3.729175	-1.420107
C	1.692442	2.548189	2.562901
H	3.217456	4.674378	1.814155

H	3.846736	5.358037	-0.496820
H	3.043619	4.027410	-2.435210
C	1.475988	1.818589	-2.415857
H	1.864825	2.224884	-3.356049
H	0.376703	1.851157	-2.478340
H	1.759477	0.757521	-2.370196
H	0.600466	2.471198	2.660940
H	2.054660	3.262020	3.311923
H	2.083853	1.555121	2.825328
H	6.379696	1.108465	2.780957
H	4.930747	1.986627	3.340058
H	5.810943	2.547550	1.891029
C	-2.735970	1.379803	-1.026116
C	-4.845192	1.685778	-2.314551
C	-6.038650	1.184167	-2.820264
C	-3.965325	0.841557	-1.620586
C	-6.382680	-0.150146	-2.609045
C	-4.314886	-0.503376	-1.420603
C	-5.524503	-0.988779	-1.898210
H	-4.583101	2.732121	-2.463592
H	-6.707526	1.840301	-3.373192
H	-7.324787	-0.535676	-2.993930
H	-5.797575	-2.025810	-1.710915
H	-3.650431	-1.151833	-0.853639
C	-1.561951	3.457563	-0.292099
C	-1.543363	4.645376	0.426083
C	-2.722512	2.667460	-0.328414
C	-2.682806	5.063601	1.112511
C	-3.872001	3.107649	0.348296
C	-3.845949	4.295050	1.067851
H	-0.679807	3.155452	-0.855475
H	-0.636438	5.247312	0.439618
H	-2.667727	5.994848	1.675472
H	-4.736389	4.619389	1.602707
H	-4.772427	2.496473	0.330097
H	0.649415	-2.058961	0.535996
N	-0.841750	-0.830849	1.263455
N	-1.604140	0.724216	-1.069591
H	-1.840155	-0.500659	-2.607888

***E*-INT7**

N	-0.719824	0.338045	1.202054
C	0.324231	-0.418055	1.018641
Pt	0.158961	-2.400970	0.690045
H	-2.007277	-2.125956	2.871948
N	-2.965685	-1.961508	2.535200
H	-2.161757	-0.505317	1.345441
N	1.575079	0.136609	1.023531
Cl	-0.062005	-4.841454	0.283365
Cl	0.250869	-2.735355	3.085054
C	0.053398	-2.063338	-1.163867

C	3.831689	0.049197	0.768390
C	6.310699	-0.171671	0.536742
C	7.446945	-0.926400	0.259104
C	5.032805	-0.748885	0.478717
C	7.330595	-2.271849	-0.077290
C	4.929383	-2.111517	0.141646
C	6.064758	-2.859447	-0.131254
H	6.417105	0.876987	0.810202
H	8.428424	-0.457858	0.312336
H	8.218821	-2.862341	-0.295345
H	5.963276	-3.912362	-0.389870
H	3.944908	-2.573690	0.098433
C	3.551437	2.114695	2.176653
C	3.926658	1.523242	0.961337
C	3.554600	3.499793	2.318927
C	4.306728	2.349035	-0.104552
C	3.927472	4.311392	1.249778
C	4.305222	3.733416	0.039262
H	3.253091	1.479452	3.011246
H	4.597141	1.897166	-1.052880
H	4.597850	4.362890	-0.799396
H	3.921517	5.394400	1.359249
H	3.255296	3.944386	3.266597
N	-0.022314	-1.824865	-2.309205
C	-0.073156	-1.536732	-3.686504
C	-0.887510	2.362173	2.533651
C	-0.821111	3.753565	2.618978
C	-0.599268	1.745276	1.300070
C	-0.489320	4.526052	1.508664
C	-0.276082	2.515241	0.161495
C	-0.226971	3.906491	0.291378
C	-1.236945	1.520719	3.721287
H	-1.032754	4.233160	3.575234
H	-0.440692	5.610584	1.591746
H	0.025234	4.505780	-0.584238
C	-0.001632	1.858608	-1.157654
H	-0.137211	2.564467	-1.985694
H	-0.667231	1.001528	-1.321574
H	1.026705	1.471952	-1.226681
H	-2.110295	0.880982	3.527911
H	-1.462118	2.139889	4.596629
H	-0.419623	0.838269	3.992099
H	-0.886392	-2.099876	-4.155080
H	0.873887	-1.817971	-4.157345
H	-0.244744	-0.464716	-3.831442
C	-3.584171	-1.512731	0.251234
C	-3.685785	-3.997435	0.288666
C	-4.346261	-5.187394	0.007925
C	-4.313601	-2.768051	0.029803
C	-5.637624	-5.164684	-0.516496

C	-5.602021	-2.752197	-0.522891
C	-6.263632	-3.947151	-0.780655
H	-2.657876	-4.022854	0.657016
H	-3.841008	-6.134164	0.189974
H	-6.155035	-6.098466	-0.729115
H	-7.269043	-3.928073	-1.196323
H	-6.083719	-1.799527	-0.739309
C	-3.525452	0.897531	-0.395440
C	-3.496019	1.889683	-1.367408
C	-3.566148	-0.456739	-0.765208
C	-3.502570	1.544546	-2.718389
C	-3.586959	-0.794585	-2.128238
C	-3.546387	0.202362	-3.095243
H	-3.546820	1.169613	0.659230
H	-3.469037	2.935499	-1.067291
H	-3.480557	2.323086	-3.479040
H	-3.550656	-0.069416	-4.149219
H	-3.610021	-1.843113	-2.421874
H	1.658951	1.141214	1.196412
N	2.695989	-0.585869	0.803733
N	-2.898863	-1.284783	1.340111
H	-3.442708	-2.853851	2.424479

***Z*-TS15**

N	0.682880	-1.026066	-0.316356
C	1.275987	0.137574	-0.434537
Pt	3.084532	0.659563	0.285259
H	-0.342515	0.603464	1.626069
N	-1.352470	0.482938	1.512999
H	-0.634486	-0.933718	0.135404
N	0.532293	1.167594	-0.939891
Cl	5.228592	1.338648	1.300132
Cl	1.841314	2.121877	1.772223
C	4.045655	-0.524675	-0.832121
C	-1.544958	1.960390	-1.477631
C	-3.882097	2.666520	-2.044614
C	-5.185237	2.359123	-2.427929
C	-2.912626	1.658131	-1.932522
C	-5.544336	1.044131	-2.710823
C	-3.287442	0.335057	-2.231708
C	-4.585485	0.032791	-2.613577
H	-3.613844	3.699590	-1.830173
H	-5.922848	3.155728	-2.509471
H	-6.563987	0.805781	-3.008457
H	-4.852126	-0.999557	-2.838902
H	-2.539722	-0.451583	-2.154318
C	-0.191662	4.055637	-1.724510
C	-1.173340	3.330934	-1.036177
C	0.219753	5.302418	-1.261280
C	-1.737969	3.879430	0.122807
C	-0.340011	5.835422	-0.102860

C	-1.319826	5.122747	0.586267
H	0.251076	3.634328	-2.627500
H	-2.494252	3.315082	0.668651
H	-1.755853	5.534483	1.494879
H	-0.010069	6.804937	0.265697
H	0.984949	5.854114	-1.804089
N	4.691222	-1.250774	-1.485508
C	5.475308	-2.159389	-2.221767
C	1.814503	-3.000299	-1.126727
C	2.497488	-4.191915	-0.874771
C	1.415423	-2.208178	-0.033247
C	2.779226	-4.591428	0.428039
C	1.669744	-2.608740	1.290229
C	2.365288	-3.803103	1.497062
C	1.537759	-2.548675	-2.527943
H	2.811795	-4.808302	-1.717976
H	3.317756	-5.519908	0.611714
H	2.583650	-4.111479	2.519551
C	1.217438	-1.779241	2.452308
H	1.666912	-2.133852	3.386065
H	0.126152	-1.818463	2.585249
H	1.492295	-0.721370	2.333754
H	0.481222	-2.287006	-2.679568
H	1.806363	-3.324217	-3.254520
H	2.103272	-1.641406	-2.785405
H	6.393915	-1.666452	-2.553945
H	4.916894	-2.507309	-3.097051
H	5.732066	-3.016514	-1.591360
C	-2.740578	-1.299217	0.870623
C	-4.078163	-1.502161	2.977252
C	-5.059884	-1.029368	3.843769
C	-3.784475	-0.800652	1.802434
C	-5.756679	0.137974	3.540192
C	-4.477334	0.379158	1.507029
C	-5.463958	0.840893	2.372622
H	-3.537538	-2.419947	3.207077
H	-5.283507	-1.579658	4.755763
H	-6.528368	0.502386	4.215748
H	-6.008076	1.752152	2.131463
H	-4.243914	0.925328	0.592800
C	-2.057103	-2.949217	-0.907299
C	-2.344911	-4.054353	-1.694258
C	-2.993989	-2.472743	0.029751
C	-3.571534	-4.709115	-1.573631
C	-4.229153	-3.134895	0.132097
C	-4.511724	-4.241873	-0.660039
H	-1.102907	-2.444478	-1.039797
H	-1.603835	-4.407861	-2.409297
H	-3.792873	-5.576128	-2.193012
H	-5.475195	-4.738066	-0.561881

H	-4.979773	-2.775711	0.831749
H	0.835232	2.114406	-0.708514
N	-0.725263	0.953530	-1.422546
N	-1.602793	-0.669443	0.806583
H	-1.815442	0.487338	2.421362

***E*-TS15**

N	0.225500	1.282004	-0.691713
C	-0.439958	0.182564	-0.433609
Pt	0.474314	-1.606659	-0.328504
H	1.883458	-0.389058	-2.657019
N	2.788761	0.042831	-2.443905
H	1.613366	1.104156	-1.031641
N	-1.783751	0.256276	-0.244570
Cl	1.646424	-3.785377	-0.251819
Cl	-0.051219	-1.887650	-2.672117
C	0.891237	-1.294698	1.487529
C	-3.826597	-0.635810	0.176442
C	-6.038264	-1.720536	0.604546
C	-6.804785	-2.828491	0.954857
C	-4.638416	-1.804220	0.551509
C	-6.190166	-4.040575	1.253621
C	-4.029615	-3.036440	0.852741
C	-4.798115	-4.138289	1.196976
H	-6.534621	-0.782833	0.360305
H	-7.889553	-2.741560	0.987808
H	-6.789493	-4.907732	1.525345
H	-4.307595	-5.083886	1.422618
H	-2.945344	-3.114750	0.806506
C	-4.376477	1.318365	-1.295560
C	-4.462978	0.693586	-0.041977
C	-4.903563	2.592063	-1.487988
C	-5.088191	1.372796	1.011561
C	-5.519749	3.260164	-0.431906
C	-5.612920	2.647377	0.816095
H	-3.887967	0.798510	-2.120392
H	-5.158203	0.896538	1.989125
H	-6.094890	3.164556	1.643850
H	-5.927691	4.258378	-0.581123
H	-4.822406	3.066489	-2.464398
N	1.154878	-1.072182	2.607523
C	1.437136	-0.824470	3.965011
C	-0.558952	3.260947	-1.872762
C	-1.142699	4.528840	-1.835161
C	-0.410608	2.552715	-0.664800
C	-1.576624	5.083058	-0.633938
C	-0.828338	3.106787	0.563527
C	-1.418075	4.374201	0.552061
C	-0.118746	2.642490	-3.162679
H	-1.265719	5.079341	-2.768296
H	-2.036205	6.070126	-0.622636

H	-1.748381	4.807102	1.496838
C	-0.637889	2.365742	1.852172
H	-0.642237	3.054006	2.705154
H	0.311045	1.813906	1.859792
H	-1.432452	1.624960	2.030553
H	0.949218	2.383335	-3.155441
H	-0.290129	3.319429	-4.006663
H	-0.656760	1.706538	-3.369036
H	2.501630	-0.989389	4.159489
H	0.848415	-1.500687	4.592461
H	1.179271	0.210964	4.210222
C	3.618568	0.777479	-0.331168
C	4.642840	-1.485809	-0.460177
C	5.751921	-2.322203	-0.404720
C	4.803117	-0.097539	-0.328408
C	7.026541	-1.784122	-0.230492
C	6.083161	0.434738	-0.127139
C	7.189823	-0.406500	-0.091068
H	3.642052	-1.913670	-0.566097
H	5.613766	-3.398327	-0.491080
H	7.893835	-2.440709	-0.195719
H	8.183016	0.014643	0.051527
H	6.205317	1.510709	-0.009644
C	2.738264	2.969791	0.471554
C	2.514607	3.888442	1.489569
C	3.409705	1.765008	0.734158
C	2.956836	3.618475	2.783787
C	3.870938	1.512150	2.036264
C	3.636470	2.430641	3.052937
H	2.418400	3.195314	-0.545053
H	1.995960	4.819793	1.268321
H	2.784316	4.340037	3.580334
H	3.985663	2.217469	4.061936
H	4.395834	0.580998	2.247928
H	-2.237335	1.165539	-0.363374
N	-2.543880	-0.823258	0.057741
N	2.692574	0.706071	-1.246192
H	3.512230	-0.673848	-2.434676

TS16

N	0.415558	1.640291	0.475495
C	0.865535	0.530322	0.365856
Pt	2.084008	-0.847987	-0.268858
H	-0.431448	-1.633659	1.020853
N	-0.482306	-0.694655	1.435235
Cl	3.581970	-2.541531	-1.145925
Cl	0.430739	-1.349943	-1.952842
C	3.406798	-0.460086	1.050485
C	-2.779084	-0.671096	0.577528
C	-3.726440	-2.638788	-0.598035
C	-3.940189	-4.006790	-0.706293

C	-2.985097	-2.114076	0.474787
C	-3.430270	-4.875935	0.256793
C	-2.476849	-3.001461	1.440634
C	-2.704744	-4.367127	1.332630
H	-4.117324	-1.964538	-1.357989
H	-4.503777	-4.397090	-1.551508
H	-3.597458	-5.947763	0.169072
H	-2.313481	-5.038463	2.094550
H	-1.928484	-2.615839	2.298790
C	-3.380010	1.546208	-0.397502
C	-3.776082	0.309253	0.145090
C	-4.326344	2.502434	-0.746638
C	-5.149697	0.049019	0.291014
C	-5.684205	2.236158	-0.583408
C	-6.090622	1.003613	-0.071800
H	-2.324338	1.740872	-0.592607
H	-5.473859	-0.902117	0.710199
H	-7.150010	0.787856	0.054572
H	-6.425042	2.981069	-0.867613
H	-3.998401	3.450799	-1.168775
N	4.227207	-0.237942	1.852142
C	5.236027	-0.030059	2.814962
C	-0.293389	3.879340	0.942151
C	-0.275832	5.232502	0.603273
C	0.449162	2.989347	0.141935
C	0.438025	5.682455	-0.502522
C	1.179452	3.420977	-0.985414
C	1.151773	4.782476	-1.288441
C	-1.093475	3.387046	2.106044
H	-0.836365	5.936115	1.217791
H	0.437776	6.741004	-0.755784
H	1.707364	5.135122	-2.156640
C	1.943632	2.453321	-1.825403
H	2.358560	2.942285	-2.712615
H	1.316762	1.614822	-2.160054
H	2.778768	2.002200	-1.271414
H	-2.061277	2.974368	1.779817
H	-1.321705	4.201577	2.801506
H	-0.577524	2.596521	2.664379
H	5.282935	-0.893763	3.484605
H	5.008669	0.868632	3.395170
H	6.199448	0.094117	2.312238
N	-1.659864	-0.172425	1.091648
H	-1.651067	0.842705	1.233446

Z-**P3**

N	-0.492819	1.585870	0.133277
C	-0.263934	0.274943	0.024681
Pt	-1.635148	-1.190856	0.020772
H	0.326916	2.182498	0.011635
N	1.022487	-0.081733	-0.155885

Cl	-3.270531	-2.992659	-0.018605
Cl	-0.122815	-2.558883	1.301001
C	-2.876569	-0.188640	-1.010936
C	3.250580	0.419009	-0.186199
C	5.633628	1.130633	-0.429759
C	6.632659	2.094657	-0.336635
C	4.309326	1.436783	-0.079915
C	6.328422	3.378780	0.105859
C	4.016100	2.737543	0.365012
C	5.014608	3.695826	0.455586
H	5.881213	0.132863	-0.787247
H	7.653279	1.838828	-0.614809
H	7.110995	4.131353	0.183951
H	4.770766	4.696222	0.809139
H	2.996002	2.979937	0.654140
C	3.156056	-1.794397	-1.374513
C	3.613312	-1.015742	-0.301796
C	3.470396	-3.148254	-1.444191
C	4.393756	-1.617985	0.694406
C	4.234069	-3.741092	-0.441155
C	4.693290	-2.973996	0.627759
H	2.559606	-1.332192	-2.161083
H	4.751553	-1.017979	1.530187
H	5.287378	-3.434103	1.415059
H	4.470521	-4.802176	-0.491630
H	3.114608	-3.741284	-2.284385
N	-3.680963	0.362351	-1.656423
C	-4.678940	1.032358	-2.391437
C	-2.212735	3.143674	-0.544758
C	-3.431074	3.776117	-0.299660
C	-1.737162	2.223546	0.406779
C	-4.156397	3.497991	0.856098
C	-2.425577	1.961167	1.602615
C	-3.653427	2.605549	1.794115
C	-1.442288	3.407169	-1.800503
H	-3.815477	4.484537	-1.033444
H	-5.110190	3.992739	1.032671
H	-4.205674	2.410165	2.712920
C	-1.890661	1.052830	2.666025
H	-2.231966	1.380409	3.654704
H	-0.795253	1.021749	2.675545
H	-2.234056	0.017227	2.533965
H	-0.468572	3.875737	-1.600976
H	-1.994291	4.077635	-2.467508
H	-1.228504	2.480839	-2.350952
H	-5.158444	0.329803	-3.079328
H	-4.229757	1.849415	-2.965021
H	-5.428434	1.438784	-1.705366
H	1.238737	-1.079214	-0.107062
N	2.023131	0.850426	-0.139669

E-**P3**

C	-3.268792	-0.224778	-1.357841
C	-3.043558	0.713550	-0.339792
C	-3.957318	0.805500	0.717712
C	-5.073787	-0.025134	0.754053
C	-5.291488	-0.952895	-0.262829
C	-4.390115	-1.048606	-1.320554
C	-1.814349	1.554760	-0.366508
N	-0.616442	1.057302	-0.255294
N	-0.530189	-0.286092	-0.054173
C	0.639049	-0.943520	0.019230
Pt	2.443205	-0.089055	-0.062900
Cl	4.665017	0.902053	-0.110880
N	0.523169	-2.265224	0.216997
C	-0.711784	-2.968794	0.407869
C	-1.174046	-3.810701	-0.617690
C	-2.389302	-4.470154	-0.420077
C	-3.122765	-4.281179	0.747007
C	-2.645287	-3.441127	1.746408
C	-1.425002	-2.774095	1.604159
C	-0.393218	-3.969138	-1.883128
C	-0.911578	-1.882305	2.691860
C	-1.914875	3.013486	-0.530130
C	-0.762157	3.820552	-0.528950
C	-0.863384	5.196189	-0.671820
C	-2.113771	5.799669	-0.824135
C	-3.260330	5.011120	-0.841607
C	-3.164058	3.630054	-0.699813
C	1.900903	1.329036	1.077505
N	1.609263	2.191162	1.812108
C	1.201398	3.260676	2.633312
Cl	3.167721	-1.900321	-1.468362
H	-2.764770	-5.127798	-1.203426
H	-4.074549	-4.793369	0.878622
H	-3.215684	-3.300361	2.663963
H	0.171863	-1.981279	2.826389
H	-1.397565	-2.112432	3.645477
H	-1.104140	-0.819229	2.481765
H	-0.197130	-2.999812	-2.361652
H	-0.928859	-4.599751	-2.599873
H	0.589617	-4.427262	-1.709126
H	0.212961	3.350061	-0.422023
H	0.039421	5.804588	-0.669507
H	-2.190137	6.879739	-0.935167
H	-4.238528	5.470550	-0.970529
H	-4.068763	3.026144	-0.725148
H	-3.789463	1.532003	1.512191
H	-5.776587	0.052612	1.581594
H	-6.162838	-1.604347	-0.229497
H	-4.551541	-1.773988	-2.115851

H	-2.558089	-0.303463	-2.181429
H	-1.396414	-0.830586	-0.005303
H	1.378554	-2.798888	0.075971
H	0.109794	3.261372	2.717642
H	1.531835	4.207313	2.194530
H	1.642340	3.152570	3.628673
